# Cell Type-Specific Pherophorins of *Volvox carteri* Reveal Interplay of Both Cell Types in ECM Biosynthesis

**DOI:** 10.3390/cells12010134

**Published:** 2022-12-29

**Authors:** Benjamin von der Heyde, Armin Hallmann

**Affiliations:** Department of Cellular and Developmental Biology of Plants, University of Bielefeld, 33615 Bielefeld, Germany

**Keywords:** *Chlamydomonas reinhardtii*, CLSM, extracellular matrix, fluorescence, green algae, in vivo, pherophorins, *Volvox carteri*, YFP

## Abstract

The spheroidal green algae *Volvox carteri* serves as a model system to investigate the formation of a complex, multifunctional extracellular matrix (ECM) in a relatively simple, multicellular organism with cell differentiation. The *V. carteri* ECM is mainly composed of hydroxyproline-rich glycoproteins (HRGPs) and there are diverse region-specific, anatomically distinct structures in the ECM. One large protein family with importance for ECM biosynthesis stands out: the pherophorins. The few pherophorins previously extracted from the ECM and characterized, were specifically expressed by somatic cells. However, the localization and function of most pherophorins is unknown. Here, we provide a phylogenetic analysis of 153 pherophorins of *V. carteri* and its unicellular relative *Chlamydomonas reinhardtii*. Our analysis of cell type-specific mRNA expression of pherophorins in *V. carteri* revealed that, contrary to previous assumptions, only about half (52%) of the 102 investigated pherophorin-related genes show stronger expression in somatic cells, whereas about one-third (34%) of the genes show significant higher expression in reproductive cells (gonidia). We fused two pherophorin genes that are expressed by different cell types to *yfp*, stably expressed them in *Volvox* and studied the tagged proteins by live-cell imaging. In contrast to earlier biochemical approaches, this genetic approach also allows the in vivo analysis of non-extractable, covalently cross-linked ECM proteins. We demonstrate that the soma-specific pherophorin SSG185 is localized in the outermost ECM structures of the spheroid, the boundary zone and at the flagellar hillocks. SSG185:YFP is detectable as early as 1.5 h after completion of embryogenesis. It is then present for the rest of the life cycle. The gonidia-specific pherophorin PhG is localized in the gonidial cellular zone 1 (“gonidial vesicle”) suggesting its involvement in the protection of gonidia and developing embryos until hatching. Even if somatic cells produce the main portion of the ECM of the spheroids, ECM components produced by gonidia are also required to cooperatively assemble the total ECM. Our results provide insights into the evolution of the pherophorin protein family and convey a more detailed picture of *Volvox* ECM synthesis.

## 1. Introduction

**Figure 1:**
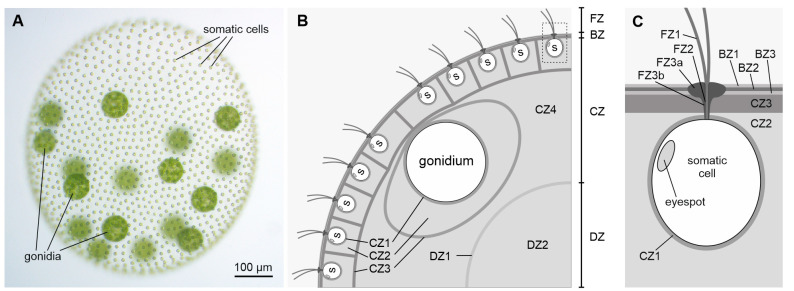
Phenotype and schematic cross section of a *V. carteri* spheroid illustrating the main zones of the ECM. More than 95% of the volume of a *V. carteri* spheroid consists of ECM. (**A**) Wild-type phenotype of an asexual, female *V. carteri* spheroid containing approximately 2000 small, terminally differentiated, biflagellate somatic cells at the surface and approximately 16 large gonidia (reproductive cells) just below the somatic cell layer. (**B**) Schematic cross section. (**C**) Enlarged section of the framed area around a somatic cell in B. (**B**,**C**) The ECM is divided into deep zone (DZ), cellular zone (CZ), boundary zone (BZ), and flagellar zone (FZ). The deep zone includes the subzones DZ1 and DZ2, the cellular zone has the subzones CZ1, CZ2, CZ3 and CZ4, the boundary zone contains the subzones BZ1, BZ2 and BZ3 and the flagellar zone includes the subzones FZ1, FZ2 and FZ3. FZ3 is subdivided into the flagellar hillock (FZ3a) and the flagellar tunnel wall (FZ3b). FZ2 is also called “flagellar collar”. The ECM structure BZ2 is conserved among volvocine algae; it is also called “crystalline layer” or “tripartite layer”. s, somatic cell.

The extracellular matrix (ECM) of a multicellular eukaryotic organism not only provides a stable scaffold that keeps cells in appropriate place and orientation, but is also involved in signaling mechanisms that regulate growth and differentiation patterns, cell homeostasis, and pathogen defense [1,2,3,4]. For the study of the ECM and other essential components required for multicellularity, the model organism *Volvox carteri* (Chlamydomonadales) and its close relatives, the volvocine algae, lend themselves as target organisms. These volvocine algae are particularly suitable for investigating the evolutionary transition to multicellular life because they diverged relatively recently from unicellular relatives, and extant species display a range of size, cell numbers and organizational complexity, from unicellular and colonial genera to multicellular genera with full germ-soma division as found in *Volvox* [5,6,7,8,9,10,11,12,13]. In the course of evolution to multicellularity, the development of a complex, multifunctional ECM from the simple cell wall of a unicellular ancestor was required. In volvocine algae, a correlation between the complexity of the organisms and the proportion of ECM per organism can be observed: A 200 nm thick ECM layer surrounds the plasma membrane of the unicellular alga *Chlamydomonas reinhardtii* (mostly called ‘cell wall‘), which accounts for about 10% of the total volume of the cell. In the multicellular alga *Volvox carteri*, ECM surrounds not only the individual cells, but the cells form a spherical monolayer that encloses ECM structures and, in addition, the cell monolayer is surrounded externally by ECM layers (Figure 1).

In an adult *Volvox* alga, the volume fraction of the ECM thus accounts for more than 95% of the organism. Despite the general simplicity of *Volvox* with only two cell types, its ECM is surprisingly elaborated, consisting of many region-specific, anatomically distinct structures, which are modified under physiological, metabolic, or developmental control [8,14]. There are four main zones of the *Volvox* ECM, the flagellar zone (FZ), the boundary zone (BZ), the cellular zone (CZ) and the deep zone (DZ), which are further subdivided into subzones (Figure 1) [14]. The FZ comprises specializations of the ECM around the flagella, with the so-called flagellar hillock (FZ3a) and the flagellar tunnel wall (FZ3b) together forming an ECM tube at the basal end of each flagellum. The BZ contains portions of the ECM that, except in periflagellar regions, are continuous over the surface of the organism and are not structurally continuous with deeper layers. The CZ1 immediately surrounds the plasma membrane of all somatic and reproductive cells. The particularly robust CZ1 around each reproductive cell (gonidium) is also called ‘gonidial vesicle’. When the gonidia begin to divide, the cell membranes detach from the gonidial vesicles, but the gonidial vesicles remain intact, and all further development to juveniles occurs inside the gonidial vesicles. CZ3 is a coherent, more fibrous material that forms honeycomb-like chambers at greater distances around individual cells. CZ2 consists of rather amorphous ECM components that fill the space between CZ1 and CZ3. CZ4 characterizes the unstructured, very loose material that lies within the somatic cell layer but outside the DZ and in which the gonidia are embedded. The DZ comprises ECM components below the CZ. DZ2 is poorly structured, fills the deepest region of the spheroid and is by far the largest region. The structure of DZ2 is similar to that of CZ4. DZ1 is a thin, fibrous boundary layer that surrounds DZ2. 

At the time when the matured juveniles hatch from their mother spheroid, they not only have to come out of the gonidial vesicle, but birth canals must also be formed through the mother spheroid [5]. To achieve this, not only the gonidial vesicle but also the ECM of the mother spheroid directly above the juveniles must be removed, while the ECM of the hatching juveniles must remain intact and completely unharmed. At least two lytic enzymes appear to be important for ECM degradation during the hatching process in *V. carteri*: VheA [15] and Lsg2 [16]. In addition, the presence of a certain pherophorin, PhS [17,18,19], in the maternal CZ1 seems to be required as an ECM plasticizer [19]. In a synergistic action, the ECM is first softened and then dissolved so that the juveniles can hatch. It has long been known that the ECMs of volvocine algae are mainly composed of hydroxyproline-rich glycoproteins (HRGPs) [20]. These HRGPs not only dominate the ECM composition of green algae, but also represent a main constituent of the ECMs of embryophytic land plants [8,18,21,22,23]. In both *V. carteri* and *C. reinhardtii*, a number of ECM components have been characterized in more detail [8,19,24,25,26,27,28,29,30,31]. Many of the ECM components characterized in *V. carteri* are HRGPs, which appear to be responsible for the assembly, architecture, and structure of the multilayered ECM [8,17,28,29,32,33,34,35,36,37,38,39]. The ECM is subject to change in the course of development. This is particularly evident during the switch from vegetative to sexual development, which is triggered by the *V. carteri* sex-inducer and leads to the synthesis of alternative ECM components [8,28,29,32,34,35,39,40,41]. Because the ECM expands dynamically and also needs to be remodeled during development, proteases and other lytic enzymes also exist in the ECM [8,27,42]. Lytic enzymes are also required for local degradation of the maternal ECM during hatching of the daughter spheroids [15,16,19]. In addition, there are enzymes in the ECM that serve to mobilize sulfates and phosphates [43,44]. Finally, defense enzymes have been found in the ECM, e.g., chitinases against fungi [45]. Furthermore, using the sequences of known ECM glycoproteins, analyses of the sequenced genomes of *V. carteri* [46] and *C. reinhardtii* [47] identified additional putative ECM proteins [19]. 

One long-known protein family with importance for ECM biosynthesis stands out among all the previous studies not only because of the number of family members: the pherophorins [17,28,33,34,35,36]. In *V. carteri*, this protein family comprises 118 members [19] including the *V. carteri* sex-inducer, which constitutes a pherophorin-related protein [34]. In *C. reinhardtii* 35 members of the pherophorin family could be identified [48]. Pherophorins typically have a dumbbell-like domain structure with two globular domains separated by a rod-shaped, highly proline-rich domain with a strongly varying length [8,17,18,35]. The prolines of the rod-shaped domain are post-translationally modified to hydroxyproline [8,28]. All pherophorins biochemically studied so far were glycoproteins, i.e., they contained covalently-linked oligosaccharide chains (glycans). The building blocks of these glycans are predominantly the sugars arabinose, galactose and mannose, indicating O-linked glycosylation of serine, threonine and hydroxyproline [8,18,28]. However, in the rod-shaped domain, O-glycosylation is primarily enabled by the abundant hydroxyprolines. The O-linked sugars of the pherophorins are highly sulfated [32,49] and unusual phosphodiester linkages between two arabinose residues have also been identified [17,36,50]. These phosphodiester bridges could be responsible for intermolecular cross-links between the polysaccharide parts of pherophorins [36]. For two purified pherophorins, pherophorin-DZ1 and pherophorin-DZ2, polymerization into an insoluble fibrous network was demonstrated in vitro [36], although the chemistry behind the polymerization remained unclear.

Among the pherophorins studied so far, distinctly different expression patterns were found. While some pherophorins showed constitutive expression from the end of embryogenesis [34,51], expression of others was strongly enhanced after induction of sexual reproduction [17,28,34,35,36] or, in others, detectable only at specific developmental stages [37]. In a whole transcriptome RNA-Seq analysis, 84% of the *V. carteri* pherophorins examined showed cell type-specific expression, and the number of cell type-specific pherophorins was similar for each of the two cell types [52]. However, all pherophorins of *V. carteri* that were extracted from the ECM and characterized, were specifically expressed by somatic cells. Thus, all previous biochemical characterizations of individual pherophorin proteins referred only to pherophorins synthesized by the somatic cell type; not a single pherophorin studied was expressed by gonidia.

The first pherophorin isolated from the ECM was SSG185 [33], although it was not clear at the time that it was a member of a large protein family. In particular, the protein chemical nature and posttranslational modifications of SSG185 were studied in detail [8,18,28,32,33,49,50,53]. In the process, it was also shown that SSG185, as with most of the pherophorins studied later, possesses a hydroxyproline-rich domain (HR domain) [33]. For in-situ immunolocalization of SSG185, the researchers used a part of SSG185 containing the HR domain for antibody production. The latter is also present in other pherophorins. Therefore, the previous, antibody-based finding that SSG185 localizes to CZ3 and forms the honeycomb-like cellular compartments of the *V. carteri* ECM [33], needs review, especially since several later publications built on this finding [5,8,17,18,35,50].

In this study, we present a phylogenetic analysis of all pherophorin-related genes found in *V. carteri* and *C. reinhardtii*. We are investigating the HR domains of pherophorins and, for pherophorins from *V. carteri*, we also disclose whether they are expressed in a cell type-specific manner and, if so, in which cell type. We then show the precise localization of two pherophorins by live-cell imaging using generated transformants that stably express fluorescence-tagged pherophorins. For this, we have selected the aforementioned pherophorin SSG185, a pherophorin that is mainly expressed by somatic cells. In addition, we localized a pherophorin that is mainly expressed by gonidia, namely pherophorin PhG. The results provide insights into the evolution of the pherophorin protein family, show distinctly different properties of soma-specific and gonidia-specific pherophorins, and provide information on how somatic cells and gonidia cooperatively assemble the ECM of *V. carteri*. 

## 2. Materials and Methods

### 2.1. Sources of Sequences, Sequence Processing and Expression Analysis

Gene, transcript and amino acid sequence information of pherophorins of *V. carteri* and *C. reinhardtii* has been published earlier [17,28,33,34,35,36,37,38,39,46,47,48]. Sequences of pherophorins are also available in the databases of the National Center for Biotechnology Information (NCBI) and Phytozome 12 [54]. The Phytozome 12 platform contains the genome versions 2.1 of *V. carteri* [46] and 5.5 of *C. reinhardtii* [47]. BLAST algorithms [55,56] were used to search for pherophorins, to do pairwise alignments and to calculate the statistical significance of matches (E-values). For several *Volvox* pherophorins with clearly incorrect gene models in genome version 2.1 of *V. carteri*, we utilized gene models from the older genome versions 2.0 or 1.0, or, if possible, we used sequences that were published in connection with the characterization of a certain pherophorin. The corresponding references are mentioned in Supplementary Table S1. 

The data used for analysis of cell type-specific expression of pherophorins originate from a previous whole transcriptome RNA-Seq analysis [52]. Mapping, data analysis, and bioinformatics are described there [52]. In this earlier analysis, the synchronized organisms were at the developmental stage in which the gonidia are just before the onset of embryogenesis. The two cell types were mechanically separated from each other and examined separately [52].

For identified pherophorins without any gene prediction in genome version 2.1 of *V. carteri*, expression values were determined based on sequence reads that were mapped to the corresponding position in the genome. The requirements for the determination of expression levels and cell type-specific expression were as previously described [52]. In short, expression analysis and visualization was conducted by using the short-read mapping analysis platform ReadXplorer 2.2.3 [57]. The ReadXplorer includes the R package DESeq [58,59,60], which was used to normalize the count data, calculate mean values (baseMean), fold differences in expression and *p* values of a test for differential gene expression based on generalized linear models using negative binomial distribution errors. The analysis of gene expressions is presented in boxplot diagrams sorted according to the cell type-specific expression characteristics of each gene. The boxes contain 50% of all sample values and whiskers represent the maximum and minimum values within the 1.5-fold of the interquartile range (IQR). In addition, median and average were calculated for each data set.

### 2.2. Phylogenetic Analysis

The protein sequences were aligned using the MUltiple Sequence Comparison by Log-Expectation program (MUSCLE) [61]. Minor manual optimization of the alignments, trimming, and management of multi-aligned data was performed using BioEdit v7.2.5 [62]. The alignments were managed and illustrated using GeneDoc 2.7 [63]. Unrooted phylogenetic trees were calculated using the PHYLogeny Inference Package (PHYLIP) v3.695 [64]. For each calculation, 10,000 bootstrap resamplings of multiply aligned sequences were generated each using Seqboot. Distance matrices using Dayhoff’s point accepted mutation (PAM) were computed with Protdist, trees were constructed using the neighbor-joining method [65] as implemented in Neighbor and a consensus tree was built using Consense. Phylogenetic trees were managed with TreeGraph2 [66] and finally drawn with iTOL 5.5 [67,68]. 

### 2.3. Strains and Culture Conditions

The female wild-type *Volvox carteri* f. *nagariensis* strain Eve10 originates from Japan and has been described previously [69,70,71]. As a recipient strain for transformation experiments a non-revertible nitrate reductase-deficient (nitA^−^) descendant of Eve10, strain TNit-1013 [72], was used. Because the recipient strain is unable to use nitrate as a nitrogen source, it was grown in standard *Volvox* medium [73] supplemented with 1 mM ammonium chloride (NH_4_Cl). Transformants with a complemented nitrate reductase gene were grown in standard *Volvox* medium without ammonium chloride. Cultures were grown at 28 °C in a cycle of 8 h dark/16 h cool fluorescent white light [74] at an average of ~100 μmol photons m^−2^ s^−1^ photosynthetically active radiation (PAR). Cultivation was performed in glass tubes with caps that allow for gas exchange or in Fernbach flasks, which were aerated with approximately 50 cm^3^ sterile air/min.

### 2.4. Isolation of Genomic DNA

The extraction of genomic DNA was as previously described [75] with minor modifications. The purity and quantity of the DNA was checked using agarose gel electrophoresis and a NanoDrop 1000 (Thermo Fisher Scientific, Wilmington, DE, USA) UV/Vis spectrophotometer.

### 2.5. Genomic PCR

PCR reactions with genomic DNA of *V. carteri* as a template were carried out using a PCR thermal cycler (Mastercycler Gradient; Eppendorf, Hamburg, Germany) as previously described [76,77,78] in order to amplify genomic regions that contain the target genes *phG* and *ssg185*. The PCR conditions were as follows: initial denaturation at 98 °C for 2 min followed by 35–40 cycles of 98 °C for 30 s, 55–60 °C for 30 s and 72 °C for 30–60 s; the final elongation step was at 72 °C for 5 min. If required, additional restriction sites were added to the 5’ ends of the primers in order to facilitate fusion of DNA fragments for vector construction. PCR products were analyzed by agarose gel electrophoresis and purified from the gel using the Gene sorb DNA extraction kit (GENOMED, Löhne, Germany) according to the manufacturer’s manual.

### 2.6. Construction of Vectors for Expression of Fusion Proteins in V. carteri

For assembly of the expression vector pPhG-YFP (Supplementary Figure S1) carrying the *V. carteri phG* gene (Vocar.0001s0298) fused to the *yfp* reporter gene, the pBluescript II SK (-) vector (Stratagene, La Jolla, CA, USA) was used as a backbone. The insert of vector pPhG-YFP consists of four parts, which were amplified by PCR using genomic DNA of *V. carteri* or plasmids carrying the *yfp* gene as a template. Appropriate artificial restriction sites were added to the PCR primers to facilitate cloning. The first part of the insert (1.0 kb) contains the promoter region and the short 5′UTR of *phG* (artificial *Kpn*I to artificial *Bgl*II). The second part (3.0 kb) begins right before the start codon of *phG*, lasts until the codon before the stop codon and includes the five introns of *phG* (artificial *Bgl*II to artificial *Spe*I/*Xba*I). The third part (0.8 kb) contains a 15 bp linker sequence, which codes for a flexible pentaglycine interpeptide bridge (Gly5), the intronless *yfp* gene (mVenus), a 24 bp sequence coding for a strep tag and the stop codon (artificial *Xba*I to artificial *Xba*I). The fourth part (1.3 kb) contains the 3′UTR of *phG* (artificial *Xba*I to artificial *Not*I).

For assembly of the expression vector pSSG185-YFP (Supplementary Figure S2) carrying the *V. carteri ssg185* gene (Vocar.0002s0564) fused to the *yfp* reporter gene, the pUC8 vector [79] was used as a backbone. The insert of vector pSSG185-YFP consists of four parts, which were subcloned or PCR amplified from plasmids containing the *ssg185* gene or the *yfp* gene. Whenever necessary, appropriate artificial restriction sites were added to the PCR primers to facilitate cloning. The first part of the insert (7.1 kb) contains the promoter region of *ssg185*, the short 5′UTR and the section from the start codon of *ssg185* until a *Bam*HI site in the seventh and last intron (*Eco*RI to *Bam*HI). The second part (0.7 kb) begins at the *Bam*HI site in the seventh intron and lasts until the codon before the stop codon (*Bam*HI to artificial *Kpn*I). The third part (0.7 kb) contains a 15 bp linker sequence, which codes for a flexible pentaglycine interpeptide bridge (Gly5), the intronless *yfp* gene (mVenus) and the stop codon (artificial *Kpn*I to artificial *Kpn*I;). The fourth part (1.0 kb) contains the 3′UTR of *ssg185* (artificial *Kpn*I to *Bam*HI). It should be noted that the *ssg185* gene in genome version *Volvox* v2.1 in Phytozome 12 [54] contained a 272 bp gap in exon 6 and the following intron 6, which we closed (Supplementary Figure S2).

The *yfp* gene used for both expression vectors was previously engineered to match the codon usage of *C. reinhardtii* [80] but also works well in *V. carteri* [19,72,81].

### 2.7. Stable Nuclear Transformation of V. carteri by Particle Bombardment

Stable nuclear transformation of *V. carteri* strain TNit-1013 was performed as previously described [82] with some modifications and by using a Biolistic PDS-1000/He (Bio-Rad, Hercules, CA, USA) particle gun [83]. Gold microprojectiles (1.0 µm in diameter, Bio-Rad, Hercules, CA, USA) were coated as described earlier [76,77]. Algae of the nitrate reductase-deficient recipient strain were co-bombarded with the selectable plasmid vector pVcNR15 [84] and the non-selectable plasmid vectors pPhG-YFP or pSSG185-YFP. The plasmid vector pVcNR15 allows for selection of transformants because it carries the wild-type *V. carteri* nitrate reductase gene (nitA), the gene that complements the mutation of strain TNit-1013. For selection of transformants, the nitrogen source of the *Volvox* medium was switched from ammonium to nitrate and the bombarded algae were then incubated under standard conditions in petri dishes (9 cm diameter) filled with approximately 35 mL liquid medium. From the sixth day on after particle bombardment, algae cultures were examined for green and living transformants (nitA^+^) in a background of numerous bleaching, unaltered organisms (nitA^−^). Each identified transformant was transferred to fresh selective medium for further culturing. Aside from the expression of nitA, expression of the co-transformed fused gene constructs was verified by fluorescence microscopy.

### 2.8. Confocal Laser Scanning Microscopy

For live-cell imaging, cultures were grown under standard conditions and examined using an inverted LSM780 confocal laser scanning microscope (Carl Zeiss MicroImaging GmbH, Jena, Germany) equipped with 63× LCI Plan-Neofluar and 10× Plan-Apochromat objectives (Carl Zeiss MicroImaging GmbH, Jena, Germany). The confocal pinhole diameter of the microscope was set to 1 Airy unit, which corresponds to an optical section of 0.8 μm. The YFP fluorescence of the PhG:YFP and SSG185:YFP fusion proteins was excited by an argon-ion (Ar^+^) laser at 514 nm and the emitted fluorescence was detected at 520–550 nm. Chlorophyll fluorescence also was excited at 514 nm and detection was at 650–700 nm. Fluorescence intensity was recorded in bidirectional scan mode for YFP and chlorophyll in two channels simultaneously. Transmission images were obtained in a third channel by using a transmission-photomultiplier tube (trans-PMT) detector. Images were captured with a bit depth of 12 bits per pixel (4096 gray levels) and analyzed using the ZEN black 2.1 digital imaging software (ZEN 2011, Carl Zeiss MicroImaging GmbH, Jena, Germany). Image processing and analysis was carried out using Fiji (ImageJ 1.51w) [85]. The lambda scan function of ZEN was used to verify that recorded signals originated from YFP fluorescence. In the lambda mode, the spectrum of the emitted light was recorded by a gallium arsenide phosphide (GaAsP) QUASAR photomultiplier detector (Carl Zeiss MicroImaging GmbH, Jena, Germany), which allowed for simultaneous 18-channel readouts. Emission spectra between 486 and 637 nm were recorded for each pixel with a spectral resolution of 9 nm using a main beam splitter MBS 458/514 and 488 nm laser light for excitation. After data acquisition, spectral analysis for the regions of interest was performed, which allowed the separation of spatially overlapping emission signals.

## 3. Results

### 3.1. Phylogenetic Analysis of V. carteri and C. reinhardtii Pherophorins

**Figure 2:**
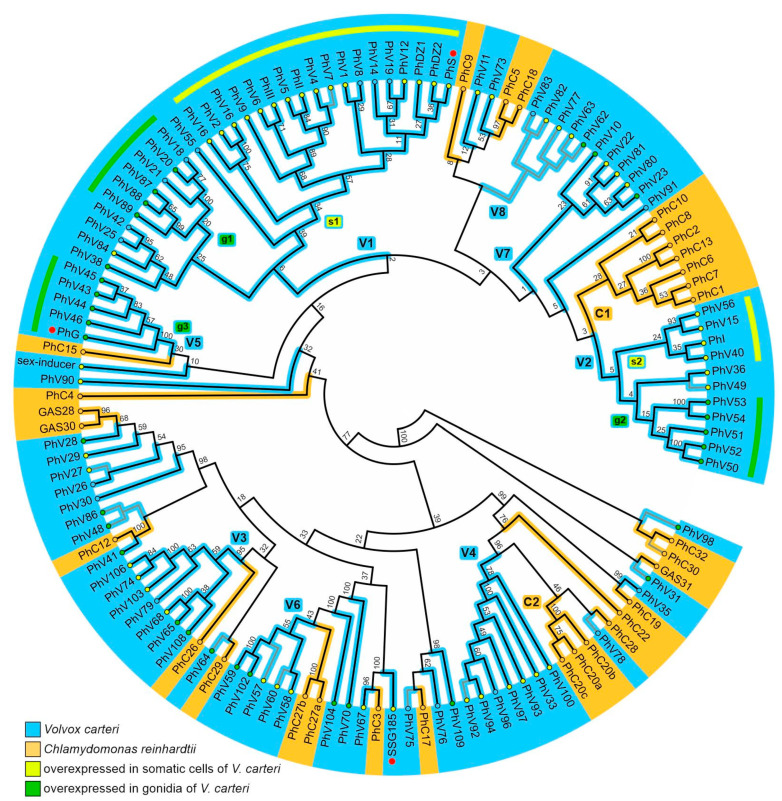
Phylogenetic tree of pherophorins of *V. carteri* and *C. reinhardtii*. Sequence relationship between the deduced amino acid sequences of pherophorins of the multicellular species *V. carteri* (blue) and the unicellular species *C. reinhardtii* (orange). Red dots indicate the positions of the pherophorins SSG185, PhG and PhS. The unrooted bootstrap consensus tree is based on 10,000 replicates calculated using the neighbor-joining method. The bootstrap values of the branch points are indicated. The tree was calculated using an alignment of the C-terminal domain of the pherophorins. However, with some pherophorins the C-terminal domains were unusable due to issues with their sequences and/or gene models. For those pherophorins, trees based on alignments of the N-terminal domains were analyzed and the pherophorins were added at the corresponding position of the tree (gray lines). Details about all pherophorins are listed in Table S1. In total, 99 pherophorins of *V. carteri* and 30 pherophorins of *C. reinhardtii* are shown. Pherophorins that are overexpressed in somatic cells (yellow-filled circles) or gonidia (green-filled circles) of *V. carteri* are highlighted. Larger sub-branches of *V. carteri* pherophorins (V1 to V8) and of *C. reinhardtii* pherophorins (C1 and C2) are indicated. Sub-branches that contain pherophorins that are overexpressed in somatic cells (s1 and s2) or gonidia (g1 to g3) of *V. carteri* are indicated and highlighted with yellow (somatic cells) or green (gonidia) arcs.

To examine evolutionary relationships and potential diversification of pherophorins, 118 previously identified pherophorin-related genes of *V. carteri* [19] and 35 of its unicellular relative *C. reinhardtii* [48], thus a total of 153 genes, were used to deduce the corresponding protein sequences. Because the N- and C-terminal domains of pherophorins are separated by a low complexity HR domain of varying length (see below), N-terminal and C-terminal pherophorin domains were aligned and analyzed separately without including the HR domain to avoid artifacts. The average length of the N-terminal pherophorin domain was 170 amino acids and that of the C-terminal domain was 155 amino acids. After trimming, alignment blocks with a length of 129 amino acids were obtained for the N-terminal pherophorin domain and 133 amino acids for the C-terminal domain. The trimmed sequences were used for the phylogenetic analysis. However, in several pherophorins either C- and/or N-terminal domains were unusable for our analysis due to issues with their sequences and/or gene models. Phylogenetic trees constructed independently with only N-terminal domains or only C-terminal domains came to nearly the same results. Because trees based on the C-terminal domain showed higher bootstrap values, further analyses were conducted using the C-terminal domains. The obtained phylogenetic tree in Figure 2, an unrooted bootstrap consensus tree, includes 99 pherophorin-related proteins of *V. carteri* and 30 pherophorin-related proteins of *C. reinhardtii*. Our analyses also includes the *V. carteri* sex-inducer, which constitutes a pherophorin-related protein [34].

One third of the pherophorin-related proteins of *Chlamydomonas* (10 of 30) and about three fourths of the pherophorin-related proteins of *Volvox* (74 of 99) cluster in species-specific sub-branches (C1 and C2, V1 to V8 in Figure 2). This indicates that the corresponding pherophorin genes arose by gene duplication after *Chlamydomonas* and *Volvox* diverged from their common unicellular ancestor. Nevertheless, the existence of quite a few sub-branches that contain both *Chlamydomonas* and *Volvox* pherophorins (Figure 2) suggests that the genome of their last common ancestor already contained several pherophorin genes.

The pherophorins of *V. carteri* were also examined with respect to their cell type-specific gene expression using data of a previous transcriptome-wide expression analysis [52]. In fact, 81 of the 99 pherophorins in Figure 2 show significant overexpression in one of the two cell types (82%). More precisely, nearly half of all pherophorins show significant overexpression in somatic cells (49%) and about one third exhibits significant overexpression in gonidia (32%). To further examine whether for *Volvox* pherophorins close relationship among each other is accompanied by the same cell type-specific gene expression behavior, significant overexpression in one of the cell types was mapped onto the phylogenetic tree of pherophorins (Figure 2). Two sub-branches with pherophorins overexpressed in somatic cells (s1 and s2 in Figure 2) and three sub-branches with pherophorins overexpressed in gonidia (g1, g2 and g3 in Figure 2) could be identified among the eight *Volvox*-specific sub-branches (V1–V8). About two fifths of the *Volvox* pherophorins (38 of 99) cluster in *Volvox*-specific sub-branches that each contain pherophorins with the same cell type-specific gene expression behavior. The remaining *Volvox*-specific pherophorins, including pherophorin SSG185, are in heterogenous sub-branches (Figure 2). Our further protein of interest, PhG, belongs to a sub-branch with pherophorins overexpressed in gonidia (g3 in Figure 2).

### 3.2. The HR Domain of Pherophorins

A clear distinguishing feature of pherophorins is the length of their HR domain. Therefore, we wanted to investigate whether there are differences or similarities between multicellular *Volvox* and unicellular *Chlamydomonas* with respect to the lengths of the HR domains of pherophorins. The lengths of the HR domains of pherophorins vary widely both in *V. carteri* and *C. reinhardtii* (Supplementary Figure S3A, Supplementary Table S1). However, the average length of the HR domains is quite different between these species: the average is 89 amino acids in *V. carteri* and 181 in *C. reinhardtii*. Thus, the mean length of *V. carteri* HR domains is approximately half that of *C. reinhardtii* HR domains. This considerable difference in length is not caused by a few extreme outliers but by an overall shift in the distribution of HR domain lengths (Supplementary Figure S3A).

For *V. carteri*, we also wanted to know whether there is a relationship between the lengths of HR domains of pherophorins and their cell type-specific gene expression using data of a previous transcriptome-wide expression analysis [52]. The group of pherophorin genes with overexpression in somatic cells show almost the same distribution of HR-domain lengths when compared to the distribution of HR-domain lengths in the group of pherophorin genes with overexpression in gonidia (Supplementary Figure S3B). However, the length distribution of these two groups with cell type-specific overexpression differs from the length distribution in those pherophorins that show similar gene expression in both cell types, which exhibit a clear shift to longer HR domain lengths (Supplementary Figure S3B). In the latter group, the share of pherophorins with HR domains of 0 to 10 amino acids is less than half of that of pherophorins with cell type-specific overexpression, whereas the share of pherophorins with HR domains of more than 250 amino acids is three or seven times higher than in the group with cell type-specific overexpression (Supplementary Figure S3B).

Because of this finding and due to the fact that the cumulative intensity of expression of pherophorins with similar gene expression in both cell types is much lower than those of pherophorin genes with cell type-specific overexpression (see below), we checked whether there is a connection between intensity of expression and the length of the HR domain. For it, we divided the pherophorin genes in groups with lower (baseMean ≤ 100) or higher expression (baseMean > 100) and analyzed their distribution within classes that were sorted according to the lengths of the corresponding HR domains (Supplementary Figure S3C). In doing so, it became obvious that the share of pherophorin genes with lower expression increases with increasing lengths of the HR domains (Supplementary Figure S3C). A similar result was obtained when we calculated the cumulative intensity of expression of pherophorins separately for the classes that were sorted according to the lengths of the corresponding HR domains (Supplementary Figure S3D). We found that about 70% of the cumulated expression of all pherophorins is accomplished by pherophorins with an HR domain of up to 50 amino acids, whereas pherophorins with an HR domain of more than 250 amino acids contribute only about 0.9% (Supplementary Figure S3D). Among the pherophorins with shorter HR domains, the biggest contribution comes from the class with an HR domain between 11 and 50 amino acids (42%, Supplementary Figure S3D). The pherophorins on which we focus in this work, PhG (Supplementary Figure S4) and SSG185 (Supplementary Figure S5), have HR domains of 14 or 40 amino acids, respectively (Supplementary Table S1). Thus, these two pherophorins belong to the latter HR domain class.

### 3.3. Expression Analysis of Pherophorin Genes of V. carteri

**Figure 3:**
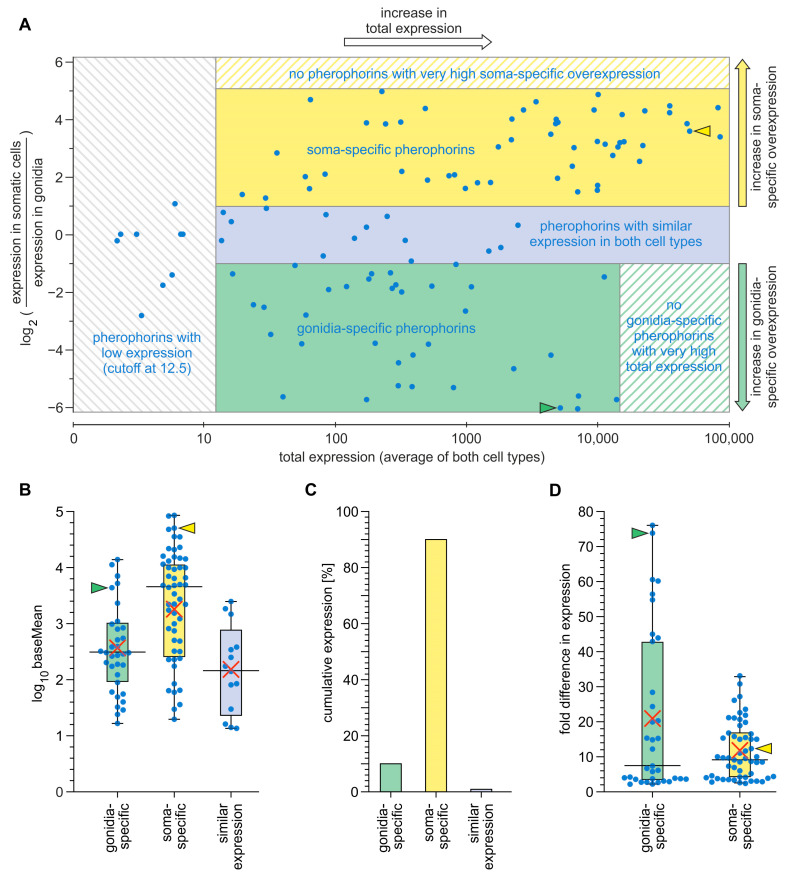
Analysis of *V. carteri* pherophorin genes concerning cell type-specific expression. The underlying data originate from a previous whole transcriptome RNA-Seq analysis [52]. A pherophorin gene is classified as being cell type-specifically expressed if the fold difference in expression between the two cell types is ≥2 and the significance value is ≤0.05. (**A**) MA-plot (Bland–Altman plot) of expression data of pherophorin genes. Each point in this two-dimensional plot shows the relationship between two sets of data: M-values (log-intensity ratios, *Y*-axis) represent the log_2_ fold difference in expression intensity of a given gene between the two cell types (somatic cells versus gonidia), and A-values (log-intensity averages, *X*-axis) represent the absolute intensity of expression (mean of normalized counts) of the same gene in logarithmic scale. Fifty-three pherophorin genes (52%) with positive M-values show higher expression in somatic cells compared to the other cell type (yellow background), 35 pherophorin genes (34%) with negative M-values show higher expression in gonidia compared to the other cell type (green background) and 14 pherophorin genes (14%) show similar expression in both cell types (light blue background). The test for differential expression was based on DEseq calculations [58] and Benjamini–Hochberg multiple testing adjustment [86]. The false discovery rate (FDR) value was set to q = 0.1. An average baseMean expression value greater than 12.5 was sufficient for robust expression analysis (cutoff at 12.5). (**B**–**D**), The pherophorin genes are sorted according to whether they are specifically expressed in gonidia (green bars) or in somatic cells (yellow bars) or whether they show similar expression in both cell types (light blue bars). (**B**) Overall intensity of expression of each individual pherophorin gene. The calculation for each pherophorin gene was (baseMean_gonidia_ + baseMean_somatic cells_)/2, which corresponds to the mean baseMean (expression average). The results are depicted as boxplots on a logarithmic scale. (**C**) Cumulative intensity of expression of all pherophorin genes that show the respective cell type-specific expression characteristics. For this calculation the mean baseMean values of the pherophorin genes shown in A were summed up separately for each category (gonidia, somatic cells and similar expression) and for all three categories together. Each category is expressed as a percentage of the total sum. (**D**) Fold difference in expression between the two cell types calculated for each pherophorin gene with cell type-specific expression. The results are depicted as boxplots on a linear scale. (**B**,**D**) Whiskers represent the maximum and minimum values within the 1.5-fold of the interquartile range (IQR). Median (horizontal line) and average (red cross) are indicated. The positions of the further characterized pherophorin genes *phG* (green arrowhead) and *ssg185* (yellow arrowhead) are indicated.

The previously identified 118 pherophorin-related genes of *V. carteri* [19,28,33,34,35,46] were subject to a joint investigation of cell type-specific expression using data of a previous transcriptome-wide expression analysis [52]. However, for 16 pherophorin-related genes, the expression level was too low for a robust expression analysis. The expression level must exceed a certain minimum expression threshold, corresponding to an average baseMean value of 12.5 [52]. The 16 genes did not meet this criterion for the developmental stage studied, where the gonidia are just before the onset of embryogenesis, and therefore could not be used here. Among the remaining 102 pherophorin-related genes are 88 genes (86%) with significant differential expression between the two cell types (fold difference in expression ≥ 2 and P_adjusted_ ≤ 0.05). More precisely, 53 pherophorin genes (52%) show significant higher expression in somatic cells compared to gonidia and 35 pherophorin genes (34%) show significant higher expression in gonidia compared to somatic cells. Only 14 pherophorin genes (14%) show approximately equal expression in both cell types. For better comparison of cell type-specific expression, total expression and expression differences between the 102 pherophorin-related genes, the expression data of the investigated pherophorin-related genes were visualized in an MA-plot (Figure 3A). The pherophorin-related genes were also sorted according to their cell type-specific expression and depicted with their respective overall expression intensity (expression average; mean baseMean) in a logarithmic boxplot diagram (Figure 3B). Details regarding each investigated pherophorin gene (e.g., genome position, expression value, fold-difference in cell type-specific expression) can be found in Supplementary Table S1. The median expression value of all soma-specific pherophorin genes is at a baseMean of 4792, whereas it is only at 302 for gonidia-specific pherophorin genes and 157 for pherophorin genes with similar expression in both cell types (Figure 3B). Thus, the median expression value of soma-specific pherophorin genes is more than fifteen times higher than that of gonidia-specific pherophorin genes and more than thirty times higher than that of pherophorin genes with similar expression in both cell types.

In a different calculation approach, we summed up the mean baseMean values of pherophorin genes separately for each category of cell type-specific expression and also for all pherophorin genes in total. This calculation approach also revealed a strong dominance of soma-specific pherophorin genes (Figure 3C): The cumulative expression of the soma-specific pherophorin genes accounts for approximately 90% of the cumulative expression of all pherophorin genes. Approximately 9% come from gonidia-specific pherophorin genes and approximately 1% of pherophorin genes with similar expression in both cell types.

For each pherophorin gene with cell type-specific expression, i.e., 88 genes in total, also the extent of difference in expression between the two cell types was investigated to reveal its specificity for the given cell type. Remarkably, the smaller group of pherophorin genes with gonidia-specific expression (35 genes) contains the genes with the highest fold difference in expression between the cell types, more precisely, the group includes the genes with the nine highest fold-difference values out of 88, which range between 43 and 76 fold overexpression in gonidia compared to somatic cells (Figure 3D, Supplementary Table S1). On average, gonidia-specific pherophorin genes show an approximately 21-fold higher expression in gonidia in relation to somatic cells, whereas soma-specific pherophorin genes exhibit only an average of approximately 12-fold higher expression in somatic cells in relation to gonidia (Figure 3D, Supplementary Table S1). Thus, there is a tendency that gonidia-specific pherophorin genes show a more distinct cell type-specific expression than soma-specific pherophorin genes.

The further characterized pherophorin gene *phG* shows a 74-fold overexpression in gonidia compared to somatic cells and it is therefore among the genes with the highest specificity for gonidia (Figure 3D, Supplementary Table S1). Our other gene of interest, the pherophorin *ssg185*, shows a 12-fold overexpression in somatic cells compared to gonidia, which also reflects the average for soma specific pherophorins (Figure 3D, Supplementary Table S1).

### 3.4. Generation of V. carteri Transformants Expressing Fluorescence-Tagged Pherophorins

Our experimental approach for a rigorous in vivo localization analysis of the pherophorins PhG and SSG185 was the generation of transgenic organisms that express these proteins with a fluorescence tag for imaging by confocal laser scanning microscopy (CLSM). This genetic experimental approach does not require extraction of ECM components, which limited previous biochemical approaches to extractable, non-covalently cross-linked, proteins.

**Figure 4:**
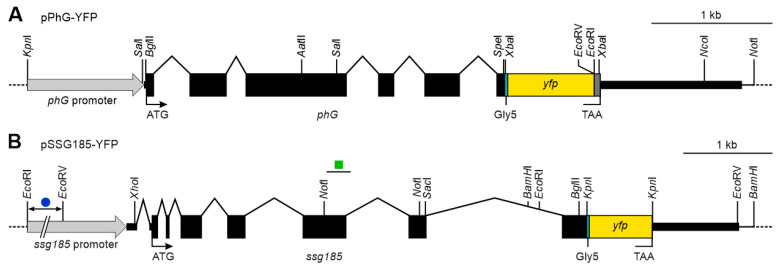
Schematic diagram of the transformation vectors pPhG-YFP and pSSG185-YFP. (**A**) Vector pPhG-YFP carries a genomic fragment of *V. carteri* genomic DNA containing the complete *phG* gene including its five introns (black), a short linker sequence, which codes for five glycines (Gly5, cyan), the coding sequence of *yfp* (yellow) and a strep-tag coding sequence (dark grey). The 5′ and 3′ flanking sequences, including the promoter region (light grey arrow), the short 5′ UTR and the long 3′ UTR, also come from the *phG* gene of *V. carteri*. (**B**) Vector pSSG185-YFP carries a genomic fragment of *V. carteri* genomic DNA containing the complete *ssg185* gene including its seven introns (black), a short linker sequence, which codes for five glycines (Gly5, cyan) and the coding sequence of *yfp* (yellow). The 5′ and 3′ flanking sequences, including the promoter region (light grey arrow), the short 5′-UTR and the long 3′ UTR, also come from the *ssg185* gene of *V. carteri*. In the 5′ flanking sequence, a 1877 bp DNA fragment between *Eco*RI and *Eco*RV sites is depicted shortened to save space (dark blue circle). The *ssg185* gene of the current genome version 2.1 contains a 272 bp sequence gap, which we closed by sequencing (green square, Supplementary Figures S2 and S7). The utilized *yfp* coding sequence has been codon-adapted for *C. reinhardtii* but it is also effectively expressed in *V. carteri*.

The first gene of interest, *phG*, contains five introns and the resulting coding sequence is 1578 bp in length (Supplementary Figures S4 and S6). The gene has a very short 5′ UTR (14 bp) and a quite long 3′ UTR (1168 bp). The second gene of interest, *ssg185*, contains seven introns, with one intron in the 5′ UTR, and the resulting coding sequence is 1455 bp in length (Supplementary Figures S5 and S7). Its 5′ UTR has a usual length (153 bp) but its 3′ UTR is quite long (891 bp). Chimeric genes were constructed that allow for expression of fusion proteins in which the C-terminus of PhG or SSG185 is fused via a pentaglycine interpeptide bridge (Gly5) to a yellow fluorescent protein (YFP) (Figure 4A,B, Supplementary Figures S1 and S2). Both chimeric genes are driven by the endogenous promoter regions of *phG* or *ssg185*, respectively, and the 5′- and 3′-UTRs also come from these genes.

Stable nuclear transformation of the nitrate reductase-deficient *V. carteri* recipient strain TNit-1013 was achieved by particle bombardment using two vectors simultaneously: one of the non-selectable plasmid vectors carrying the *phG* or *ssg185* gene, respectively, fused to the *yfp* reporter gene (vectors pPhG-YFP or pSSG185-YFP) and the plasmid vector pVcNR15 as a selectable marker. The obtained transformants were investigated for stable genomic integration of the DNA constructs and for expression of the desired proteins at sufficient levels by performing a fluorescence microscope-based screening using an LSM780 confocal laser scanning microscope.

### 3.5. Development-Dependent In-Vivo Localization of the Gonidia-Specific Pherophorin PhG

Under the light microscope, the phenotype of *V. carteri* transformants expressing the chimeric *phG*:*yfp* gene under control of the endogenous *phG*-promoter is indistinguishable from that of wild-type *V. carteri* algae. The same applies for the growth and development of the transformants. Thus, the genetic modification did not affect the appearance or fitness of the algae in a detectable way. Synchronous cultures of PhG:YFP-expressing transformants were grown under standard conditions in an 8-hour-dark/16-hour-light cycle, which results in a life cycle of 48 h. Because pherophorin PhG has never been examined before, we investigated all developmental stages throughout the life cycle of *V. carteri* by CLSM.

**Figure 5:**
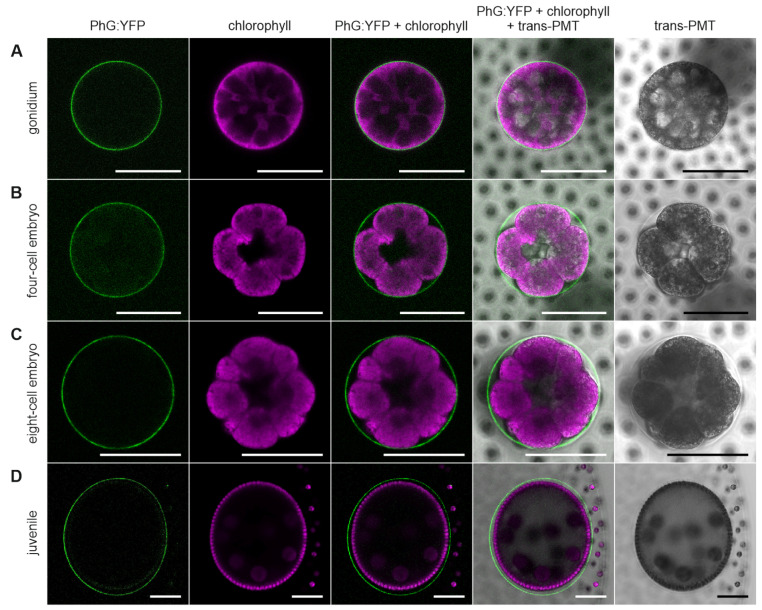
Localization of PhG:YFP in *V. carteri* progeny before, during and after embryogenesis. Transformants expressing the *phG*:*yfp* gene under the control of the endogenous *phG* promoter were analyzed in vivo at different developmental stages for the localization of the PHG:YFP fusion protein. CLSM optical cross-sections of progeny that are in their natural situation inside their parent. Transmission-PMT (trans-PMT) images are included for orientation. (**A**) Mature gonidium shortly before the first cell division. (**B**) Embryo at the four-cell stage. (**C**) Embryo at the eight-cell stage. (**D**) Juvenile after inversion, shortly before hatching. Column 1: YFP fluorescence of the PhG:YFP protein (green), detected at 520–550 nm. Column 2: Chlorophyll fluorescence (magenta), detected at 650–700 nm. Column 3: Overlay of YFP fluorescence of PhG:YFP protein (green) and chlorophyll fluorescence (magenta). Column 4: Overlay of PhG:YFP fluorescence (green), chlorophyll fluorescence (magenta) and transmission-PMT (trans-PMT). Column 5: Transmission-PMT alone. Scale bars: 50 µm.

In the life cycle, the fluorescence signal of the PhG:YFP fusion protein appears for the first time shortly before onset of embryogenesis, when the maturing gonidium prepares for its first cell division (Figure 5A).

**Figure 6:**
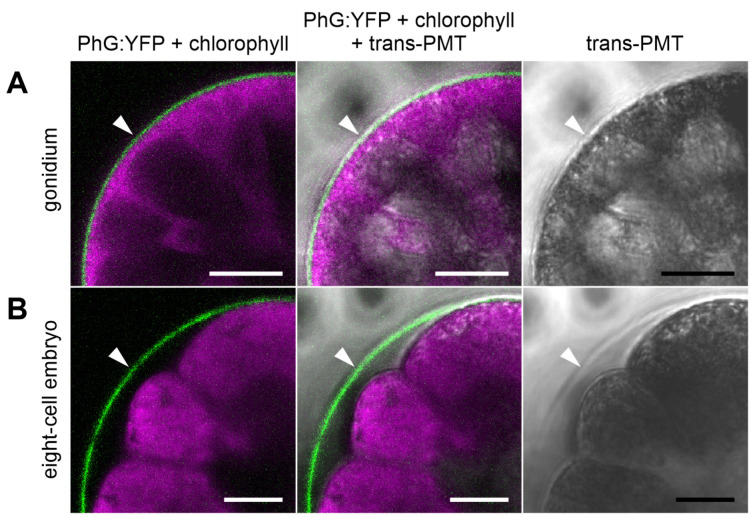
Closer examination of the distance between the PhG:YFP-stained gonidial vesicle (CZ1) and enclosed cells. Transformants expressing the *phG*:*yfp* gene were analyzed in vivo at different developmental stages for the localization of the PHG:YFP fusion protein and the relative position of the enclosed cells. High-magnification CLSM optical cross-sections focusing on the gonidial vesicle (arrowhead) and the cell borders. Transmission-PMT (trans-PMT) images are included for orientation. (**A**) Mature gonidium shortly before the first cell division. There is no detectable space between the gonidial vesicle and the plasma membrane of the gonidium. (**B**) Embryo at the eight-cell stage. The dividing cells detach in the most places from the surrounding gonidial vesicle. The distance between the gonidial vesicle and the plasma membrane of the cells is up to 8 µm. Column 1: Overlay of YFP fluorescence of PhG:YFP protein (green) and chlorophyll fluorescence (magenta). Column 2: Overlay of PhG:YFP fluorescence (green), chlorophyll fluorescence (magenta) and transmission-PMT (trans-PMT). Column 3: Transmission-PMT alone. Scale bars: 12.5 µm.

The fluorescence-tagged pherophorin is found as a thin layer at the outer surface of the gonidium in close proximity to the cell membrane (Figure 5A). Obviously, the ECM zone CZ1 of the gonidium (Figure 1B), also called gonidial vesicle, is formed at this time. The developing gonidial vesicle with PhG:YFP seems to be in direct contact to the plasma membrane of the gonidium (Figure 6A). It is, in fact, the only place where PhG:YFP can be detected. During the subsequent process of embryogenesis, PhG:YFP continuously can be detected in this layer around the developing embryo (Figure 5B,C). The layer thickness of the gonidial vesicle is always approximately 1 µm on the basis of the PhG:YFP fluorescence. In the course of embryogenesis, the dividing cells detach in most places from their surrounding gonidial vesicle (Figure 6B). The distance between the gonidial vesicle and the plasma membrane of the cells even reaches up to 8 µm (Figure 6). After completion of cell divisions, the embryo turns itself inside-out in a process called inversion and then grows in size inside its mother spheroid. In the developing juveniles, the PhG:YFP fluorescence is still located at the gonidial vesicle (Figure 5D). However, PhG:YFP fluorescence intensity decreases as development of the offspring progresses to juveniles (Supplementary Figure S8). In the juvenile stage, the fluorescence then decreases rapidly and finally disappears by the time the juveniles are released.

### 3.6. Development-Dependent In Vivo Localization of the Soma-Specific Pherophorin SSG185

As for PhG:YFP transformants, the phenotype, growth and development of transformants expressing the chimeric *ssg185*:*yfp* gene under control of the endogenous *ssg185*-promoter is indistinguishable from that of wild-type *V. carteri* algae. Therefore, this genetic modification did not affect the appearance or fitness of the algae in a detectable way. Synchronous cultures of SSG185:YFP-expressing transformants were grown under standard conditions in an 8-hour-dark/16-hour-light cycle, which results in a life cycle of 48 h.

**Figure 7:**
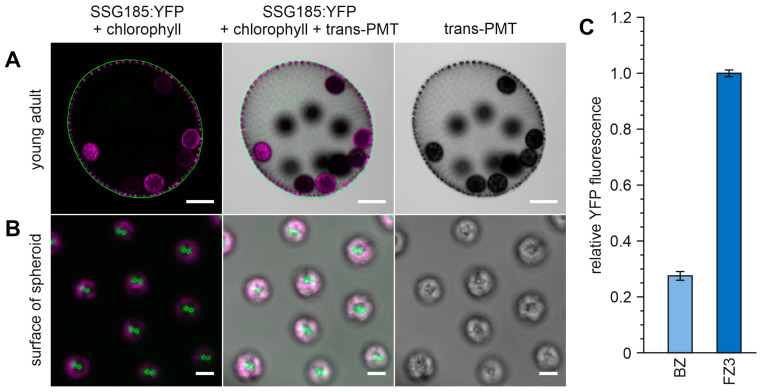
Localization of SSG185:YFP at the surface of young adult *V. carteri* spheroids. Transformants expressing the *ssg185*:*yfp* gene under the control of the endogenous *ssg185* promoter were analyzed in vivo for the localization and fluorescence intensity of the SSG185:YFP fusion protein. (**A**) CLSM optical cross-section of a young adult spheroid with mature gonidia shortly before the first cell division. Note that the continuous layer of SSG185:YFP at the surface of the spheroid shows increased fluorescence right above each somatic cell. Scale bars: 100 µm. (**B**) High-magnification CLSM top view onto the surface of the spheroid. Note that the two fluorescent spots above each somatic cell have a non-fluorescent center, which corresponds to the position of the flagella. Due to the viewing direction and CLSM settings, weaker fluorescence on the entire surface (as shown in the cross-section in A) cannot be observed here. Scale bars: 5 µm. (**A**,**B**) Column 1: Overlay of YFP fluorescence of SSG185:YFP protein (green) and chlorophyll fluorescence (magenta). Column 2: Overlay of SSG185:YFP fluorescence (green), chlorophyll fluorescence (magenta) and transmission-PMT (trans-PMT). Column 3: Transmission-PMT alone. (**C**) To measure fluorescence intensity, it was quantified in the YFP channel (520–550 nm) in a straight line spanning through the BZ or FZ3, respectively, and the maximal value was recorded. The recorded intensity values were normalized to the highest recorded value in order to exclude the influence of differing laser intensities between the replicates. The data represent three biological replicates with three technical replicates each. Error bars indicate standard deviation of the mean of the three biological replicates.

We first identified the fluorescence-tagged pherophorin SSG185:YFP in CLSM optical cross-sections of young adults where it covered the entire outer surface of the spheroids with a continuous thin layer (Figure 7A). This layer has a thickness of approximately 0.8 µm. A closer look reveals that the fluorescence constitutes no perfectly even and uniform sphere-shell. Instead, there are little spots with stronger fluorescence intensity exactly above each somatic cell where the flagella protrude (Figure 7A). In a top view onto the surface of the *Volvox* spheroid, the spatial location of these small fluorescent spots becomes even clearer: Significant amounts of pherophorin SSG185:YFP are concentrated in two tiny circles around the flagella basis, right at the place where the flagella emerge from the somatic cells (Figure 7B).

**Figure 8:**
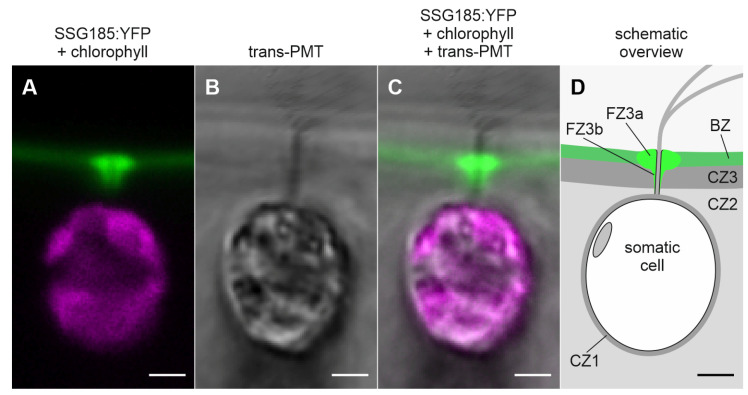
Localization of SSG185:YFP at the flagellar region of the somatic cells. Transformants expressing the *ssg185*:*yfp* gene were analyzed in vivo for the localization of the SSG185:YFP fusion protein. High-magnification CLSM optical cross-section focusing on the flagellar region of somatic cells. Transmission-PMT (trans-PMT) images and a schematic overview are included for orientation. (**A**) Overlay of YFP fluorescence of SSG185:YFP protein (green) and chlorophyll fluorescence (magenta). (**B**) Transmission-PMT (trans-PMT). (**C**) Overlay of SSG185:YFP fluorescence (green), chlorophyll fluorescence (magenta) and transmission-PMT. (**D**) Schematic overview indicating the position of SSG185:YFP and of the involved ECM zones. For unknown reasons, the trans-PMT (**B**,**C**) shows interference effects that cause extra lines near the surfaces of the spheroid and the flagella. These lines should not be considered. BZ, boundary zone; CZ, cellular zone; FZ, flagellar zone; FZ3a, flagellar hillock; FZ3b flagellar tunnel wall. Scale bars: 2 µm.

The center of each circle does not show any notable fluorescence because it is occupied by a flagellum. A side view of a somatic cell under high magnification provides further information about the exact localization of SSG185:YFP (Figure 8). Under these in vivo conditions, the flagella beat with a frequency of approximately 25 Hz [87] but a high-resolution CLSM scan takes not less than a few seconds. Therefore, the freely moving part of a flagellum always looks blurry due to its continuing beating during the image capture process, whereas the stiffened part of the flagellum, which goes through the ECM, appears as a straight black rod (Figure 8B). The fluorescent SSG185:YFP protein is located in the outermost part of the ECM, the boundary zone, and covers the entire outer surface of the spheroid. Furthermore, SSG185:YFP is found at even higher concentration in specialized areas of the boundary zone around the flagella exit points, the flagellar hillocks (FZ3a) and the flagellar tunnel walls (FZ3b) (Figure 8). There is no notable fluorescence in the lumina of the flagellar tunnels because they are occupied by the flagella (Figure 8). Our quantification of fluorescence revealed that the intensity of SSG185:YFP fluorescence both at the flagellar hillocks and the walls of the flagellar tunnels is approximately 3.8 times higher than that in the boundary zone (Figure 7C).

**Figure 9:**
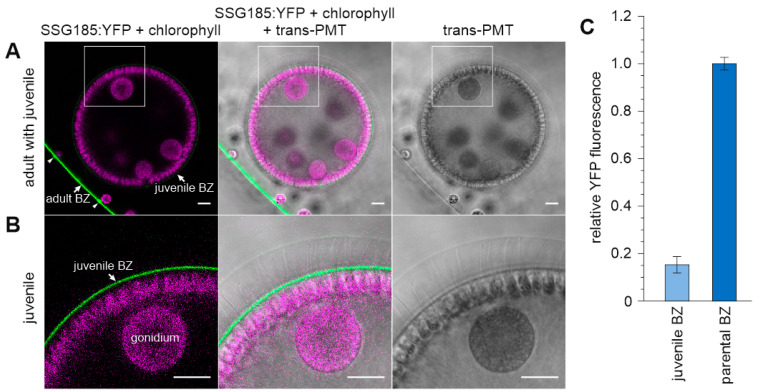
Beginning accumulation of SSG185:YFP at the surface of juveniles after embryonic inversion. Juveniles of transformants expressing the *ssg185*:*yfp* gene were analyzed in vivo for the localization of the SSG185:YFP fusion protein. The juveniles are located inside their parental spheroid and their developmental stage is 1.5 h after embryonic inversion. The boundary zones (BZ) both of the adult and the juvenile are indicated. Transmission-PMT (trans-PMT) images are included for orientation. (**A**) CLSM optical cross-section showing a part of an adult with one of its juveniles inside. Note that the YFP signal intensity of SSG185:YFP is much stronger in the adult BZ than in the juvenile BZ. Furthermore, the YFP signal can be found at the flagellar hillocks of the adult (arrowheads) but not at those of the juvenile. The latter becomes clearer in B. (**B**) Magnified detail of the framed area in A. It should be emphasized that the YFP signal in B was amplified with higher gain than in A. Column 1: Overlay of YFP fluorescence of SSG185:YFP protein (green) and chlorophyll fluorescence (magenta). Column 2: Overlay of SSG185:YFP fluorescence (green), chlorophyll fluorescence (magenta) and transmission-PMT (trans-PMT). Column 3: Transmission-PMT alone. Scale bars: 10 µm. (**C**) Comparison of the YFP fluorescence intensity in the BZ of juvenile algae 90 min after embryonic inversion with the parental BZ. Fluorescence intensity was quantified in the YFP channel (520–550 nm) in a straight line spanning through the corresponding BZ and the maximal value was recorded. The recorded intensity values were normalized to the highest recorded value in order to exclude the influence of differing laser intensities between the replicates. The data represent three biological replicates with three technical replicates each. Error bars indicate standard deviation of the mean of the three biological replicates.

We also investigated all the other developmental stages throughout the life cycle of *V. carteri* for SSG185:YFP fluorescence. The earliest sign of fluorescence was detected in young juveniles about one hour after embryonic inversion of the developing embryo. For comparison of fluorescence intensities in the surrounding ECM of juveniles with that of adults, we examined young juveniles inside their parents (Figure 9). In one place, the cell layer with the somatic cells of the juvenile is quite close to the cell layer with the somatic cells of its parent, which allows a good comparison of both layers. The somatic cells of the juveniles are very close to each other with only little ECM material between the cells, whereas somatic cells of adults are quite far apart from each other with vast amounts of ECM between them. At this developmental stage, the measured intensity of SSG185:YFP fluorescence in the boundary zone of adults is approximately six times higher than that in the boundary zone of young juveniles (Figure 9C). In the boundary zone of the parental spheroid, the flagellar hillocks and the walls of the flagellar tunnels show stronger SSG185:YFP fluorescence than the rest of the boundary zone, just as described above. In contrast, in the boundary zone of young juveniles the distribution of SSG185:YFP fluorescence resembles a perfectly even and uniform sphere-shell. This becomes even clearer at higher magnification and with higher amplification of the SSG185:YFP fluorescence (Figure 9B).

**Figure 10:**
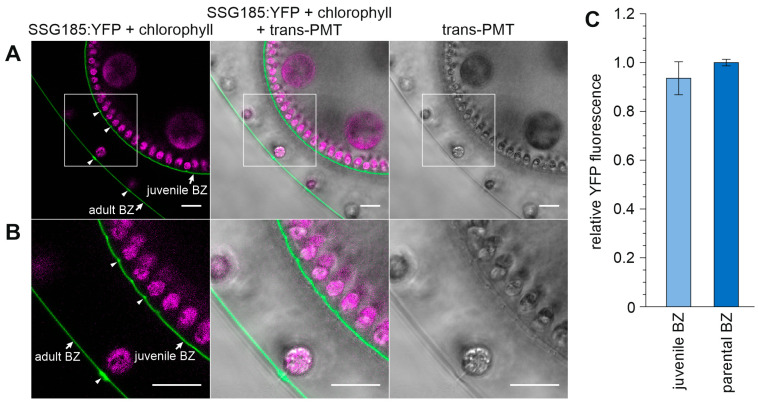
Localization of SSG185:YFP at the surface of juveniles shortly before hatching. Juveniles of transformants expressing the *ssg185*:*yfp* gene were analyzed in vivo for the localization of the SSG185:YFP fusion protein. The juveniles are located inside their parental spheroid and their developmental stage is shortly before hatching. The boundary zones (BZ) both of the adult and the juvenile are indicated. Transmission-PMT (trans-PMT) images are included for orientation. (**A**) CLSM optical cross-section showing a part of an older adult with one of its juveniles inside. Note that the YFP signal intensity of SSG185:YFP in the adult BZ is similar to that in the juvenile BZ. Furthermore, the YFP signal can be found at the flagellar hillocks of both the adult and the juvenile (arrowheads). This becomes clearer in B. (**B**) Magnified detail of the framed area in A. Column 1: Overlay of YFP fluorescence of SSG185:YFP protein (green) and chlorophyll fluorescence (magenta). Column 2: Overlay of SSG185:YFP fluorescence (green), chlorophyll fluorescence (magenta) and transmission-PMT (trans-PMT). Column 3: Transmission-PMT alone. Scale bars: 15 µm. (**C**) Comparison of the YFP fluorescence intensity in the BZ of juvenile algae shortly before release with the parental BZ. Fluorescence intensity was quantified in the YFP channel (520–550 nm) in a straight line spanning through the corresponding BZ and the maximal value was recorded. The recorded intensity values were normalized to the highest recorded value in order to exclude the influence of differing laser intensities between the replicates. The data represent three biological replicates with three technical replicates each. Error bars indicate standard deviation of the mean of the three biological replicates.

The parental situation with stronger SSG185:YFP fluorescence at the flagellar hillocks and the walls of the flagellar tunnels gradually develops in juveniles and it progresses quite far, even before the release of the juveniles from their parental spheroids (Figure 10). In juveniles, shortly before release, the measured intensity of SSG185:YFP fluorescence in the boundary zone has already risen to the same value as in the boundary zone of adults (Figure 10C). Additionally, the condition that flagellar hillocks and the walls of the flagellar tunnels show stronger SSG185:YFP fluorescence than the rest of the boundary zone is the same as with their parents (Figure 10). However, one difference is that the parental flagellar hillocks are about 3.5 times larger in diameter than those of the juveniles shortly before release, which indicates that the flagellar hillocks are continuously growing during the whole life of the organisms.

## 4. Discussion

### 4.1. Not Only Somatic Cells but Also Gonidia Synthesize Pherophorins

**Figure 11:**
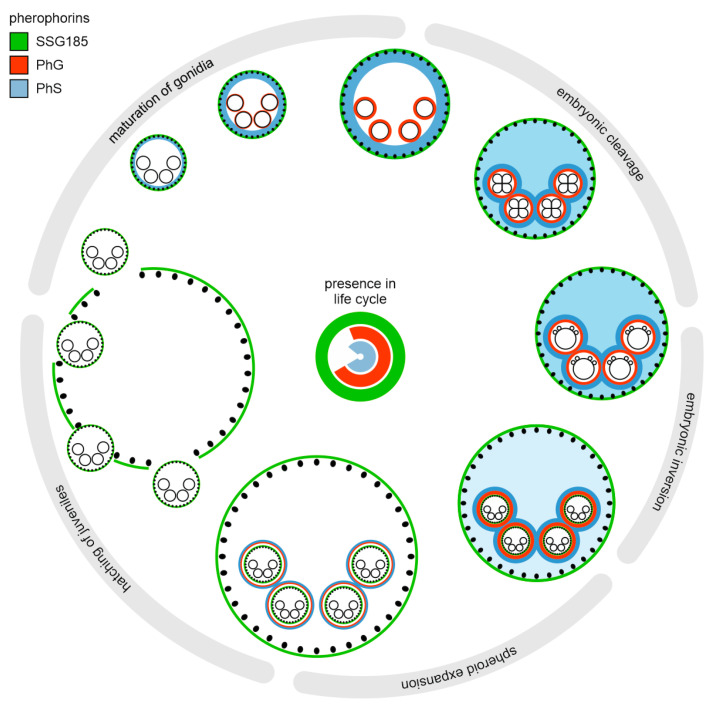
Schematic representation of the localization of three pherophorins, PhG, SSG185 and PhS, in the life cycle of *V. carteri*. The localization of three pherophorins, PhG, SSG185 and PhS, is known throughout the vegetative life cycle of *V. carteri*, which lasts 48 h under standardized laboratory conditions. The ECM synthesis begins after completion of cell divisions and embryonic inversion. At about the beginning of ECM biosynthesis, pherophorin SSG185 (green) is synthesized by somatic cells and can be detected in the boundary zone and at the flagellar hillocks. SSG185 remains detectable there throughout the life cycle and it can therefore be found in both the parent and the post-inversion juveniles. In the maturation phase of gonidia, the maturing gonidia start synthesis of PhG (red), which is subsequently detectable in the gonidial vesicle. Shortly before hatching of the juveniles, the gonidial vesicle is enzymatically degraded and PhG is no longer detectable. PhS (blue) is initially secreted into the CZ of the somatic cells, where it does not remain [19]. As ontogeny progresses, it increasingly diffuses into the inner ECM layers of the spheroid and it eventually accumulates around the gonidial vesicle [19]. During hatching of the juveniles, PhS is released.

The transition from unicellular to complex, multicellular organisms appears to be closely linked to the evolution of an elaborate, multifunctional ECM from the simple cell wall of a unicellular ancestor [12]. Through close microscopic examination of the *Volvox* ECM, its complex, anatomical structure is known [14], but unanswered questions remain concerning the detailed molecular composition and function of components. It has long been known that the ECM of volvocine algae consists mainly of HRGPs [20] and it also became clear early on that a specific family of HRGPs, the pherophorins, is critical to the ECM [17,28,33,34,35,36]. Some HRGPs of *Volvox* were studied biochemically [8,17,18,28,32,33,34,35,36,49,50], although it has always been assumed that all ECM components are synthesized by somatic cells. A later transcriptome-wide expression study showed that there are also pherophorin genes that are specifically expressed in gonidia [52]. Within the framework of this work, it has now been possible to localize a gonidia-specific pherophorin protein, PhG, in vivo (Figure 5, Figure 6 and Figure 11).

Accordingly, both somatic cells and gonidia contribute to the assembly of the total ECM of *Volvox carteri*. The cumulative expression of the soma-specific pherophorin genes accounts for approximately 90% of the cumulative expression of all pherophorin genes and approximately 9% come from gonidia-specific pherophorin genes (Figure 3C). The gonidia are indeed larger than the somatic cells, but a *Volvox* spheroid has only about 16 gonidia and about 125 times as many somatic cells. Therefore, the contribution of the few gonidia appears to be quite remarkable. Nevertheless, the cell type-specific shares of the total expression of all pherophorin genes suggest that somatic cells actually produce most of the total ECM material of the spheroid. With the exception of PhG, the synthesis of all ECM proteins studied in more detail to date, such as ISG, DZ-HRGP and the pherophorins SSG185, PhS, PhI, PhII, PhIII, PhV1, PhV2, PhDZ1 and PhDZ2, has been attributed to somatic cells [17,19,28,33,34,36,37,38,39]. However, these ECM proteins have been found in or extracted from the extracellular space, more precisely the ECM zones FZ, BZ, CZ and DZ, but were not investigated with regard to cell type-specific synthesis. Therefore, the involvement of gonidia in ECM synthesis cannot be excluded on the basis of these earlier publications and, thus, the cell type of synthesis of all previously investigated ECM components needs to be (re-)examined. For the pherophorin SSG185 studied here, it has now been shown that it is mainly synthesized by somatic cells (Figure 11). While soma-specific pherophorins (such as SSG185), which have the highest proportion of cumulative expression (Figure 3C), appear to be important for the structure of the ECM throughout the spheroid except directly at the gonidia or embryos, gonidia-specific pherophorins (such as PhG) may be responsible for ECM zones around the gonidia or embryos. The latter pherophorins could form a defensive barrier for the developing next generation until hatching.

### 4.2. PhG Is a Building Block of the Gonidial Vesicle

The CZ1 around each gonidium, developing embryo and juvenile, the gonidial vesicle, seems to provide a robust protective cover against bacterial or eukaryotic invaders and other unfavorable influences that can penetrate to it through the parent. However, the gonidial vesicle must still be able to expand during maturation of the gonidium inside and, after completion of the subsequent cell division phase, provide enough space when the embryo turns its multicellular cell sheet inside out during inversion [88]. The ECM network around the gonidia appears to be very tightly meshed, as investigated chemicals such as the DAPI dye could not enter the interior of the gonidial vesicle [19]. To allow juveniles to penetrate the gonidial vesicle during hatching, the specific hatching enzyme VheA is produced [16], which is able to digest the gonidial vesicle but not the ECM of the juveniles inside the gonidial vesicle [16]. The building blocks of the vesicle must therefore be different from those of other ECM structures. This gives rise to questions such as: (i) What are the building blocks of the gonidial vesicle? (ii) Which cell type synthesizes the building blocks of the gonidial vesicle? The pherophorin PhG is almost exclusively expressed by gonidia (Figure 3D, Supplementary Table S1) and the in vivo localization analysis demonstrates that the PhG protein is localized in the gonidial cellular zone 1, the gonidial vesicle (Figure 5 and Figure 11). The observed vanishing of the PhG:YFP fluorescence during further development coincides with the degradation of the gonidial vesicle at the beginning of the hatching process (Figure 5D). These findings suggest that PhG is indeed a building block of the gonidial vesicle (Figure 11). Since a total of 35 pherophorins have been identified that show gonidia-specific expression at the mRNA level [52], it is likely that not only PhG but the entire vesicle is synthesized by the gonidium. This in turn raises the question of how a single, specific enzyme, VheA, can open the gonidial vesicle. The enzyme could either attack a common motif of many or all pherophorins in the vesicle or it attacks a specific cross-linking pherophorin, cleavage of which then disintegrates the network. In future studies, it would be interesting to identify other pherophorins of the gonidial vesicle and search for molecular similarities between them. Comparison of those pherophorins in the gonidial vesicle with pherophorins elsewhere in the ECM may also help to elucidate the molecular causes of the robustness of the gonidial vesicle.

### 4.3. SSG185 Is Located in the Boundary Zone and FZ3 Where It Appears Soon after Embryonic Inversion

While the gonidial vesicle provides a robust protective layer for the developing offspring inside the parent, the boundary zone presumably represents an outer protective barrier for the entire parental organism. The boundary zone also appears to be critical in defining the overall shape of spheroids, ensuring also proper orientation of the somatic cells and providing a basic scaffold to establish the entire ECM [37,38]. The boundary zone is probably the first ECM layer formed during ontogeny. At the very beginning of ECM formation, which starts after the completion of embryogenesis, the ECM protein ISG establishes an initial framework [37,38]. It is crucial that the construction of the functional ECM then proceeds rapidly, because a few hours after embryogenesis the cytoplasmic bridges between the somatic cells are broken down, and the ECM must then take over their task, and not only hold the cells of the organism together but also keep them in place without twisting, otherwise the spheroid will not be able to swim directionally later on [38,89]. A pherophorin that also appears very early in development is PhS [17,18,19]. In the phylogeny, PhS is located in the *Volvox*-specific sub-branch V1 (Figure 2) and therein in a sub-branch with pherophorins overexpressed in somatic cells (s1 in Figure 2). Matching the phylogenetic position in a soma-specific sub-branch, PhS is synthesized by somatic cells and then secreted into their CZ (Figure 11). However, PhS does not remain at this place: as ontogeny progresses, it increasingly diffuses into the inner ECM layers of the spheroid and eventually accumulates around the gonidial vesicle (Figure 11) [19]. Our in vivo localization of pherophorin SSG185 reveals that it is part of the BZ and the FZ3 (Figure 8), where it appears very early, specifically only 90 min after embryonic inversion (Figure 9 and Figure 11). The SSG185 forms a continuous layer at the outer surface and appears very prominently where the boundary zone is, which is why it could be the main component of the boundary zone (Figure 11). While initially also a relatively small amount of SSG185 is found in the FZ3, more and more SSG185 is accumulated there during the life cycle (Figure 9 and Figure 10). Finally, in an adult spheroid, the concentration of SSG185 in FZ3 is as much as 3.8 times higher than in BZ (Figure 7C). The significant strengthening of the FZ3 structures may suggest that the beating flagella in mature *Volvox* algae require robust, tubular ECM structures at their bases to withstand mechanical forces.

In contrast to our results, an earlier immuno-localization suggested that SSG185 is localized mainly in the honeycomb-like structure of the CZ3 surrounding the somatic cells [33]. In the corresponding photomicrograph, however, it can be seen that the BZ was also detected without this being mentioned in the publication. For this immuno-localization, the researchers used the antibody mAb 302/1 and mechanically disrupted, formaldehyde-fixed spheroids. At that time, it was not known that there are many other pherophorins in the *Volvox* ECM besides SSG185. The antibody was raised against a protease-resistant part of SSG185 carrying the HR domain, which is a typical feature of most pherophorins. Most probably this antibody, which is no longer available, targeted not only SSG185 but also other members of the pherophorin family.

Our results also suggest to adjust the ECM nomenclature. The localization of SSG185 in the BZ and FZ3 shows that these contiguous ECM zones share similarities in their composition. Because of this chemical relationship between BZ and FZ3, FZ3 should neither be assigned to FZ nor considered a specialization of CZ [14]. Earlier assignments were based only on fine structures in electron microscopic studies [14] and the components of the ECM zones were not examined.

The BZ is known as the only ECM structure conserved in volvocine algae, which include *C. reinhardtii*. In addition, the FZ in *V. carteri* and *C. reinhardtii* seem to be similar in structure and function. Since SSG185 is a component of the BZ and the FZ3, there should be a corresponding functional homolog in *C. reinhardtii*. Our phylogenetic analysis of pherophorins (Figure 2) suggests that pherophorin C3 (PhC3, Cre06.g278162) of *C. reinhardtii* is this functional homolog. In the course of a large-scale expression analysis of all three genomes, pherophorin C3 was shown to be permanently expressed throughout the life cycle of *Chlamydomonas*, with a particularly high expression just after the onset of the light phase [90]. In the light phase, cell growth takes place and the volume of the cell increases significantly, which also requires a strong ECM expansion. In fact, about 30 min after the onset of the light phase, the expression of pherophorin C3 increases to a level four times higher than its average expression level [90]. We therefore hypothesize that the function of pherophorin C3 in *Chlamydomonas* is similar to the function of SSG185 in *Volvox*. Both proteins might have the function of building up the conserved structure of the boundary zone as well as to reinforce the area around the flagella so that the ECM can withstand the mechanical forces during flagellar beating.

## 5. Conclusions

Somatic cells most likely produce the major part of the ECM of the *Volvox* spheroid, which is responsible, among other things, for the correct shaping and adaptability of the organism. However, ECM production by gonidia is also crucial. Gonidia form their own specialized ECM envelope within the mother spheroid, which provides a particularly protective, tight barrier during embryogenesis. The pherophorin SSG185 produced by soma cells is a basic structural component of the BZ and FZ3. It contributes to the outer protective layer of the organism. In addition, SSG185 forms tubular ECM structures around the flagellar bases. There, these structures presumably serve as reinforcement so that the ECM can withstand the mechanical forces during flagellar beating.

In contrast to previously characterized ECM proteins, the gonidium-specific pherophorin PhG is an ECM component produced by the gonidium. It surrounds the gonidium as a component of the gonidial vesicle. PhG thus contributes to the protective, tight barrier around the developing offspring inside the parent.

Our results provide insights into the evolution of the pherophorin protein family and convey a more detailed picture of *Volvox* ECM synthesis.

## Data Availability

All data generated or analyzed during this study are included in this article and its supplementary information files. Plasmids are available upon request.

## Supplementary Materials

The following supporting information can be downloaded at: https://www.mdpi.com/article/10.3390/cells12010134/s1: Supplementary Figure S1: Sequence of the chimeric gene *phG:yfp* as it is present in the vector pPhG-YFP; Supplementary Figure S2: Sequence of the chimeric gene *ssg185:yfp* as it is present in the vector pSSG185-YFP; Supplementary Figure S3: HR domains in *V. carteri* and *C. reinhardtii* pherophorins; Supplementary Figure S4: Schematic structure of the *phG* gene, *phG* mRNA and pherophorin-G protein; Supplementary Figure S5: Schematic structure of the *ssg185* gene, *ssg185* mRNA and SSG185 protein; Supplementary Figure S6: Genomic sequence of the *V. carteri phG* gene; Supplementary Figure S7: Genomic sequence of the *V. carteri ssg185* gene; Supplementary Figure S8: Quantification of PhG:YFP fluorescence intensity in the gonidial vesicle before, during and after embryogenesis; Supplementary Table S1: Expression characteristics of *Volvox carteri* pherophorins.

## Abbreviations

BZ	boundary zone
cDNA	complementary deoxyribonucleic acid
CLSM	confocal laser scanning microscope
CZ	cellular zone
DZ	deep zone
ECM	extracellular matrix
FZ	flagellar zone
HR	hydroxyproline-rich
HRGP	hydroxyproline-rich glycoprotein
IQR	inter quartile range
MBS	main beam splitter
mRNA	messenger ribonucleic acid
PhG	pherophorin-G
RT-PCR	reverse transcription polymerase chain reaction
SSG185	sulfated surface glycoprotein with a mass of 185 kDa
trans-PMT	transmission-photomultiplier tube detector
UTR	untranslated region
UV	ultraviolet
YFP	yellow fluorescent protein (mVenus)
