# Supplementary material for: Cell Type-Specific Pherophorins of Volvox carteri Reveal Interplay of Both Cell Types in ECM Biosynthesis
Source: Cells. 2022 Dec 29;12(1):134. doi: 10.3390/cells12010134 (PMC9818292; doi:10.3390/cells12010134)
Supplement: Supplementary file 1 [file cells-12-00134-s001.zip › Supplemental Figure S5.pdf]

Supplemental Figure S5

Schematic structure of the *ssg185* gene, *ssg185* mRNA and SSG185 protein

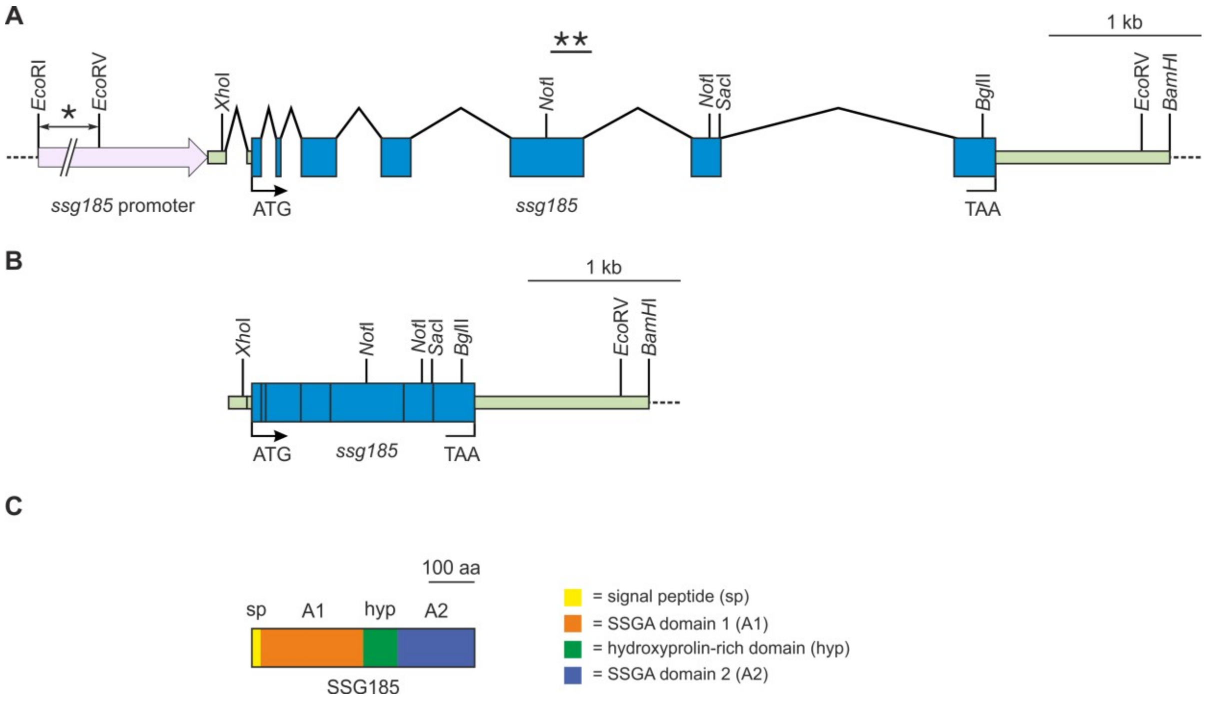

A The sequence schematized here corresponds to the 8.8 kb genomic fragment utilized in plasmid pSSG185-YFP. The *ssg185* gene is located on scaffold 2 (nucleotides 4191410 to 4198020) of the *V. carteri* genome version 2.1 (Prochnik et al., 2010) in Phytozome 12.1.6 (Goodstein et al., 2012) and has the gene ID Vocar.0002s0564. The start codon is located on the plus strand at nucleotide positions 4191698 to 4191700. The gene structure is indicated as follows: Coding sequences are represented by blue squares, intron sequences by carats, UTRs by green bars and the promoter region as used in the vector pSSG185-YFP by a pink arrow. Start (ATG) and stop (TAA) codons are highlighted. **B** Structure of the *V. carteri ssg185* mRNA. Sequence features are as indicated in A. The coding sequence (blue squares) totals 1,455 nucleotides. The 5' UTR is 153 bp in length, while there is a quite long 3' UTR of 891 bp. The complete mRNA is 2,502 nucleotides in length. **C** Structure of the *V. carteri* SSG185 protein. The polypeptide comprises 485 amino acids. As SSG185 is an extracellular protein, it possesses a cleaved N-terminal signal peptide (sp) of 19 amino acids. The mature protein comprises three domains: an N-terminal A domain (A1), a hydroxyproline-rich domain (hyp) in the middle (Hallmann, 2003) and a C-terminal A domain (A2). The hydroxyproline-rich domain consists of 81% (hydroxy) prolines.

References

Goodstein, D. M., Shu, S., Howson, R., Neupane, R., Hayes, R. D., Fazo, J., Mitros, T., Dirks, W., Hellsten, U., Putnam, N. et al. (2012) Phytozome: a comparative platform for green plant genomics. *Nucleic Acids Res.* **40**, D1178-1186.

Hallmann, A. (2003) Extracellular matrix and sex-inducing pheromone in *Volvox*. *Int. Rev. Cytol.* **227**, 131-182.

Prochnik, S. E., Umen, J., Nedelcu, A. M., Hallmann, A., Miller, S. M., Nishii, I., Ferris, P., Kuo, A., Mitros, T., Fritz-Laylin, L. K. et al. (2010) Genomic analysis of organismal complexity in the multicellular green alga *Volvox carteri*. *Science* **329**, 223-226.
