# Supplementary material for: Cell Type-Specific Pherophorins of Volvox carteri Reveal Interplay of Both Cell Types in ECM Biosynthesis
Source: Cells. 2022 Dec 29;12(1):134. doi: 10.3390/cells12010134 (PMC9818292; doi:10.3390/cells12010134)
Supplement: Supplementary file 1 [file cells-12-00134-s001.zip › Supplemental Figure S6.pdf]

Supplemental Figure S6

Genomic sequence of the *V. carteri phG* gene

TTGCCCTTCCGAAGCGGCAGGAAGCTACCGACCTGAACATGCTTTTAGGACTTTCAATCTGTGCACCC
GAACACACAAGTTACAATCAACGCTGTAATGCGGGTAAGTTAATAATTAATGCATTTGTCAAGCAATC
GGTGTTTGACATCCGTGATAAAGTCTCTTCAGAATCTGCAGCCCGAGTGTTCAATTTGTTCATTAAAA
CTTGTGAAAAGTGTATGAATGTGTCTATTTGGCATGTACTTCTGTGTTAATGATACAGCCGGTGATAC
GCGTTTGATACCACTCGTTATCCGGAACAATCGACCCGTATCGACATGATGTAGATGTCCTGGGATCG
CTGATGGATTCCCAAAAATTCTTCTGTTGCTTATCTTCAGAACAAAGTACTTCAATCTTCTATGCTAG
AAATACCAGTGCTCAACGAATGCTCCTGCCTGCCGCCACCACATCTCGCTGTATGTGTCGCAAAGGCG
GGATCCGCATATTGAACAACACCTTTCTCAATTGACTCCAAGCACCAATGTTGCCAATATATTTATTC
AGACATTGCGATATATACTCCAAGGCAAGGAAACGAACGGCATCAATGTCAAGTGCGCTAATGCACGC
ACGGGTCGCGAGCGCTACAATGCTGACGAGCCCATACTTCAATCAGGAGTAAGCGACGAAAGTGAGGA
ATATCGACTAAGCGATGACAGTGAGAAATCGTTAATTGTGCATAACAAGCGTGATTCCCGCTTGAAGG
GTTGTAGCTGCATCTAGCTTTTTGCCCGTGGCAGCAGATGGCAAATATCTACCATCGGCTGTCAAGTA
AATACGGTTTTGTCGGGGATTTAAAGTAACGAAAGGTTCAAAGGAACGGAAGGAGCTACACATTGGGC
TGCACGCGACTGATTTCAACCAGCTCCATTGTCGTACTGTACGTTCAAAGTAGTCTGAAGTAAAGCTG
ACTATAAATACGCGGTTGCCAATTATAC**GTCGAC**ATACTAATTGGGCACATAGAGGGCCTAC**ATG**CGA *Sal*I, **start**
ACACCAATGAGGAATATACTGCTGGCTGCTCTTTGCGTAGTCGCCGTATTCTCGTAAGTGTCGTTGCT
TACCTTGCTAGGGCCAGCTGCAGGCTTCGATGTACAAGCATGTAGGGTTCGCAGGCTTATTCTTAAAC
GTTTACCTACCTTCTGAGCCATGAGGAGTCGTAGCTTTAATAGCCATGCTGCAATCCGTTTGGCTTAC
GTCGCTTTGGCGGGCACGGGCGCCAGTATCACCCCGTGTCGTTGATCGCTGCGGCTCCCCTCATTTTG
CGAAATTCACCATCATATTTGTAAATGATTGATGACTTGCTGAATCTCTTGCCACCTTGCGCTTATTT
TCCAATCTGCAGGGCGTCGCACGCGCTGAGTAGTCCGCTGTCGCCCCCGCCGACAAGATACCAGGCAT
TTCCTTACTGCGCAAAATGCGTCGCTTTGGAGCCTATATACCGTCTAAAGCCTGATTTTGCCGGATTT
GGAAATCGCACCTTCTGCTTCACGCTGGAAGTGAATCCTCTCAATGCCGACTTCATGTGCAAGAATGC
ATTAATCTACAAGATGGATGTGAGTATATATCAATTGAACATGTGTTGATGTAGCGCAACTAGCACTT
CGGGCGGTTCTCGAAATCTAGCAGCCTTTCGCGTTACGGTTGTCGAACGTTAAAGCTTTAGTCAGTCA
CGAGGGCGCGCAACTGTGTGTCACTCCATTCGAGCAGATTTACCTTGCCTGGTTGTCTTCATGACATA
GAACATAAGAAGGGTTATCTACTTCCATTTTTATTATCGTTAACAGCGTTCCCCGGCGAACCCGCAGA
TGCTTCTCAACAAAGGGTGCGGAAGGGATGTTAACGTCAAGGCTTTCGTAAATGGCGTCTTCACAAGG
AGCTTCACAGAATCTCCACGTTCTGCCGCAAATGGGACTGTGATATTGAAACTGAACGATCTGACCGC
GCGACACCTGGGTATCAACAGCAATGGCGCCAGGATTTGCCTTACTATATTGAATGGGGGCAAGGGCA
ACTGCACCACTTTGAGGTCGCTCTGTGCAGACCCTAAGACCACCAACTTCAGAAAGGATAACAACGGC
ACTTACAGAAATACAAATTCCCAAGAGTGCATGGTTGCGCTATTCGCCGGGATCGACAGTGGCTCCGA
CCGTACCTGCTGCGACCCCGGTTCTCCCCCATCTCCAACGCCGCCCCCGCCTCCTCCAGGGAATTGCA
CGGAGGAGAAGGTCTGTGTCTATCTGACCTTTGGGTTCCGCGAACCG**GACGTC**GCCTTCCCCAACGGC *Aat*II
ACCTGCAATAATTTGGCCAGCCAGATACAGTTCGACCTCCTCAACTCAAGCGCATCCTACATCTTGCA
GCCCCCGGTAGTTCCACAGATCACTTGCACAGCAGACTTTATCAAAGTTTGCTTAAATTTGGACTTGC
ACGCTAACGCTACAGAGCTGCATTCCGTGGTTGGTGAAAATGCCGGTCCCTGGCTGAATTACGCTCAC
AACTCCACGTGTGCGGCAAGTCGCTCCAACTACAGCCTCACAATTACGGTCGGAGGGGATGGCGACTG
GTCGACTGGCAACGTCTCGTTCCGTTGTTTCAATGCGTCAACTGAAACCAAGTGCCAAGGGCCCTCGA *Sal*I
AGAAATCATGCAAGTGAGTCGAGCAAACCTGGAGGAGGGGGACAAGTATTGCGTGGCGGTGGGGAGTA
AACATGTCTGGCGTTGATCTAATGTGGGATTGCATGCTCGACGCGGATGCATGCACGCATCCACGTAC
GTGTCAGCAGCTACATCTTCCTGAGCTACTGGTTTGAATTGGATTTTAGCAGCTTTGTGCCCTCACAT
GATTATCCTTCCAGCTGTATCCCTCTGCTCTCGCCACCTATCCCTTTCCCTCCTTTCCTCTCCTTCCT
CCGCAGCTGCAATATGGCGAGGAACATCACCCCCTTCGCCGCCTTGCCGACTATGAGCGGTCCCTTCA
CGGGCCGCAAGAACACGAAGCTGTACTGCTTCAACATCACCGTCGTGAAGCCCATCGAGCCAAATGTG
GGTCGCAGCGCTCGCTGGCGCCGCCGTACACCGCATGATAGCATGTGCATGTCCTGGTTGATGTATGT
ATGTATGTATGTGGCCATCTACTGGCTGCGACACGAGCACAGTAGTAGTGCCACATTGACGGCTACAT
CGATTACGCCTGTACAGATGTCTAAATGGTGCATGCGGCCACATCTTCCTCCAGTAGCACTCCATTAC
GGTTCCCTTGCTATCCAATCCTATCCCTCGCATATCCCCCCTTTACAGAGCACCTGCGGTAAAAGCAA

TGTGCTCGATAAGGTCATCATCTGGGCCAACGACACCAGTTTTTACCGCAGCAGCATCAAGTCCATCG
CTCTCTACGCTGCGGGCGACACCACCGCGAAGTATGTCCCGCCGAGCTGGAACTCGGATAGACAGGAG
GTGAAGGCAACCAAGATTGGCTGGACTAAGGATAAGGCGAACGGCGGCGCCATCTGCCTGGAGCTGGA
CCAAAACGTCAACCTGGCTGACTTTTGCATATGGCGGGGTCCCAAGCAGAAGAACTCTTGCATGTAAG
GGCCTGGGGATTTATTTGGGGGTTGCCTGCATGCTTGTCATGCAGTGTGCATGCATGGCGGGATGTGA
GTGTGCGCGAACTAGGCGGCACTCGGTCGCCATCCCGGCTGCAGAAATGCAGGCATGCACCTAGCTAG
ACTATTTACGCGTGCTTCCTTGCACATGGTTGAAGAGCTCTCCAATGGCACCCTAGCCTTGCACTAGC
TTAGCTTTTCTACTGCTTAGAGGCGCCTCTGGGCCGAGCGTCCTGTCTACCTACCTTATTGTGCGTCC
CCCATGCCCATGCCCATCTTGCTGAATGCAGGGTTTTACTTTTCGATACATCCTATAGATGCTGCCCG
ATGTACCGGCCGATGGCGCTTACTACG**TAG**ATGCGTGCCGTCACAGCTGGCTAGTCTTGTGCAGAGCA **stop**
TGCACGTTGGATCACTGACCTGGTGAGGCGAGGAAGCCCCATCTAAGGTTGTGCGATTGGGCGATTTG
TGTGTTGCGTGGTAGGATGTAGAATATTTGGTTTGCGAATGTGCTTTGGATAAAATCCATGTTGGTAG
TTCCTAAACTTGTGCACGATGCTGCCCGTGAACAGGGCAGGTAAGGTTGTGTGTTTGGAAGTGTTTGG
AATTACATGGAGGTTATCTCTAAGTTTCAGTGATTAACAGTGCTTAAACAGTCATGTCTGGTTTGCGG
CTGTTTGCGTGTTCCTGCCTGTTCTTACCGGCCGTGTTGCCTGCTGGACCTGCCGCTGCTGCGCAGCC
TACCCGTGCGATTTCGCTAGGAAGAATTTCATTTCGTTGTGCAGGTCGCCTTTCTTACTTTTTCAATT
CTGTCTGTCAGAGGTGCTTGCTTCCTTGCAGCAGGTGGAACTGATCACCTCGCCGCCGTTTCGATCAT
TTAAATACCCCCTACGTCGCGCAATGATGTGGGGTATCGTAGGCTGCTTTATGCAGCCTCGTTCGCTT
ATTTGATCGCACCTGCGATTGTAGCAGCTTCATTCATTAGCTGAATCAAGTGAGCGAGCACGCTCGGT
CGGTCAGGTTTTATAACATGCTGCTTGCTGCTCAAGCTTTTGCTAATATTGTATAGCGCAGGTACGCT
TTGCTGATTGCTTCTGGGGTCGCCTAGTTCGGGGCTACTGCTTGCATGCTGTGTGTGGTGAGATAGCA
GGCTGGCTGCCATTACTGCGGAGTACGCAGCTCTTCAGCCATATATTCATAGTTAGGACGAATTGATT
AGCCAGC**CCATGG**CGATGTCAGTTCAGTTCAATGTCCATTTTCATACGCGCCTTGCGTTTTGTTTATG ***Nco*I**
GCTGTCAGTCTGCGTGATCACCTTATGTTAGATCGATGCATCCTGCAATCGTTTTGTGCGTTGTAACA
CTGCGAGCGCGACAGGCAGAGTGTTAGGCGTATTGGCGAGAAATGTCCTGTTCGGTTAGGGCGGTTTG
GTTAGGGCAGTTTCTAAAGATCCTCTGCCGGCTGATGGAAATATCGGTGTTTATGTGTAGGAAATTTC
TCCCGCCCGCCATCCATCCCCAACATTACCGTGTAACATAATCCAACAATCCTTGGCCCATCCTATCC
TATCCAAGCAGGTCTTCAAGCATACTTTTTCATCTCCTTCACACTGCAGCCGGCAGCAGGCATGATTC
CTGCGAAC

The sequence depicted here corresponds to the 5244-bp genomic fragment utilized in plasmid pPhG-YFP. The *phG* gene (former common name *phV47*) (von der Heyde and Hallmann, 2020) is located on scaffold 1 (nucleotides 2224937 to 2230180) of the *V. carteri* genome version 2.1 (Prochnik et al., 2010) in Phytozome 12.1.6 (Goodstein et al., 2012) and has the gene ID Vocar.0001s0298. The start codon is at nucleotide position 2225951-2225953 on the forward strand. The gene structure is indicated as follows: Coding sequences are shown with blue background, UTRs with green background and the promoter region with grey background. Start and stop codons are highlighted (violet font). The 5' UTR is just 14 bp in length, while there is a quite long 3' UTR of 1,168 bp. The restriction sites that are shown in Supplemental Figure S4A and B are marked (bold, underlined).

References

Goodstein, D. M., Shu, S., Howson, R., Neupane, R., Hayes, R. D., Fazo, J., Mitros, T., Dirks, W., Hellsten, U., Putnam, N. et al. (2012) Phytozome: a comparative platform for green plant genomics. *Nucleic Acids Res.* **40**, D1178-1186.
Prochnik, S. E., Umen, J., Nedelcu, A. M., Hallmann, A., Miller, S. M., Nishii, I., Ferris, P., Kuo, A., Mitros, T., Fritz-Laylin, L. K. et al. (2010) Genomic analysis of organismal complexity in the multicellular green alga *Volvox carteri*. *Science* **329**, 223-226.
von der Heyde, B. and Hallmann, A. (2020) Targeted migration of pherophorin-S indicates extensive extracellular matrix dynamics in *Volvox carteri*. *Plant J.* **6**, 2301–2317.
