# Supplementary material for: Cell Type-Specific Pherophorins of Volvox carteri Reveal Interplay of Both Cell Types in ECM Biosynthesis
Source: Cells. 2022 Dec 29;12(1):134. doi: 10.3390/cells12010134 (PMC9818292; doi:10.3390/cells12010134)
Supplement: Supplementary file 1 [file cells-12-00134-s001.zip › Supplemental Figure S7.pdf]

## Supplemental Figure S7

### Genomic sequence of the *V. carteri ssg185* gene

**GAATTC**ACATGGCGAAGTATAAGCATTGGATGGGGCTGGCGGCCGAGCAAGCGGGCAGGAAGGAGGTG *Eco*RI
CTCCCGCACATCACAATGTTTATCCCACGTTCGGCGAATTGCAAGCTTATGCAGTTTAGGCTTTGCTG
TTGGGCACTGGCGGCTAACAGAGCGCGTAAGGGACGGCTTTATATACCTAGGGAGGAGCGAGTATGCC
CCTGCTGCGGCACTGATAGCCACATCGAGGATGAGAAACACATTTTGCTGGACTGTCCGGTTTACGAC
GGAATCAGATCTGATATGGGCTTTCCACGCTTCGAACATATGAAGGACGCTATGAAGTGGGAACCTGG
ACGCCTGGCAAGAATGCTGGAACGTATATGGTCTCACCGCAACTCTATCGTTCCCTTTGGTCGTTAGT
ATACTTCATTACTTGTTTCGTTGGAAGTGCGAGGTAGGATGGTGCTTATAGGCGATTTGGCAGCCCTT
GAGCCCTCGGCTCGTACTAGGGTGGTTTGCAGGTACGGGGAACGCACCCACGACCATGTAACAACAAC
AACAATACATACGTGCCGTCTTCCCTGAAGCATATTTTCCCCCACGATGAATCGAATTATGACAAAGT
CAATATCAGGTGAACCATACGAACGGCGCCTCTTCGTGATGGGGCGACGCAGTGACTTGATCCGCCGA
GGGAAACGCCACCCCATAGAACGCCTGCAGAAGCTACAGCACGGCGGGCCGTAAAATGGAGTGTGGTT
TTGGTCCCCGACAACGATTACATTGACATGCAAGCAGCGGGTTGATTGCAAAGCTCAAGTTCATGCGA
CATCATCATTTGTATTTGACGCACATCGTGGCCGTTTTCCCCTCCTGAGGAATGAGAACGATCAGGAA
GCAAGCACGAAACTGACGCTGACTGTTGTTGCTTGCGGGTGCGAGTCAGCAACACATGCAACCCCAGT
TCAAGGGTCCAATTATGAATGCGGGGATGGGGTGGTAGGCGCGGCAGCGAGTATGCGCGTATCGGCGC
CCGGCTGGGCATCAGCCTGTTTGAGACGCCGTGCCATGCCGCTCCGCGCGGGTGCTTCGTTGGTCCAT
ACGTCTCGACTGGAATGTGTGTAGTCACAACTTCTGCCACATTCGATCTCCTTGGTCTTTGTAACACT
TTTGGGCGAGCAAATTTTAATCACTTTTTGTTGCCAAAGCGAAGGTATGGACTGGGCTTTCTAAGTCC
CGTTTCAAAAGCACATCTTTCGGAAGAAAAGGATACGAAAAGTTTTCATTTACGGCCGCATGTGCTAT
GTGGGTAGATTTTGCAGTGGCAGTTCCGCAATTCGACAACATGCCTTAGAAAATAAAATTAAGCTCCA
AGCCCAGGGCCGAAATAGAATACTGTCTATGTCTCGTGCAATCACCGGCAATTGAAGGAAGCAAGAAG
CTATTGAAATTGACCCTGAGATTCCTTTGAACGACTTATTCTCGATTGTTACGGCAGTCGCCCACGTG
AAACTGCCGCCGTTCATCAGCAGTCAGGGCAAGCTAGCGTGAAAAACAGTAACGCGCCGTATCAGTAG
TACATGTTTGAACGGCGTTTTGCTTGGATAAAATTATGAAGTTTTCTTTTGGCAAGCAGACCTTTGGT
GCATCACCCTTCTTTATAAGCAATTCATCGCTTGCAACGCACATACTAGCCAACTTCTTTTAGCGATA
TACTTATTGGTATTGTTCTAGTACTTTCTATGAACGTCGACTGTGTCCAATTGTGTTCAACTAGGATT
AAGCGTGCCACGTCAGCAGATAGACTGCTAGTACTGCTGTTCTCCCGTGTATGACTACGGTACTCTGA
AAAGGCGTTAACGCCTCATATTAACGTAAAGGTAACGTA**GATATC**AGTTGCTAAAATTACCTAGCATT *Eco*RV
AGATTATGTTGCAAAACGGATCAATACGTATCGGATAGGGATGTGGCGAAATTGCACGACGCTCGGAA
TGAAAGGTAGAATGCGTAAATGTTCTTACCTTTCCCTAGACTGCGAAGAAACAAGCAAATGATGACTT
GATCTATTTATTTTATCCAAAGGCGCCAATATCCGACGTCACATCAACCGAGCACTCCATCCAAGTTA
GCCATTGAAGCAGGAGCAGCAGCACAACAACGTAATCACCTATGAGGATATAAGTCTCATCATATTTT
AAACTATATTATACTGCACTCAGGATGACTGCCGGAAGGGAAGTGAACCGCCGGCAATCTTTACGAAG
TAGCTGGCCAAATCCATAAGCCTCATGAAATTATTAAATCAAATTAACTCTGATTAAGTTTAACTTCA
TCGCGGATGTCACCGCGTACGTTGGACTTGTCGTTACCCATCAGCATTGCTTACTGTTAACTTGGCGG
AACGTTACAATCACATCCCAGGAGGCCACCATCCTCAATATACTCTATTTTATGGTGTTTTAAGCACA
TTAGTTGGCACGCTCGCCTTCTTTCCCGCGCTCGCGCCCACCGCTCTGTACACCGTCTGGACATGCCG
TACATTATTCGCCAGGGAGGGCGCGTTGCAAGCCGTCATAGAAGGATCGGGTTCTGCGATTGCATAAT
CGTAGCTGCACCCTTACAGGCTGAAGAGTTACGGCCTCGTGGGTTGCATAATTGGAATCCCTTTTTCA
CGAAAAGGCGTGGAGTGGTCCGTTATTCCATTTCTGTCACGA**CTCGAG**ACTTGGCTACGTAAGTCTGC *Xho*I
ATGTGATGGATTCACGCGACATCGCTAGATTGTTTGATGTTGCATAATTGGCCAACATCTCAGCATTT
TAGCTGGAGAGGGTAGCTGGCAGACGCTTATCATGTCGCGGTTCCTTCCGTGCGCAGTGGTGCTCACC
GGGACCAATTTTATACGCAAAG**ATG**TCTAAGCTGCTGCTCGTGGCGCTTTTTGGCGCCATAGCAGTGG **start**
TTGCTACGAGTGCTGGTGAGTGCGCTGCCTCCTTCGACCCTTACGATGCAACGTAAATTTTCGCTACC
TGAGTTAACCTGCTTCTTTCTGCCTATTCTTTTGCTTATAACAGAGGTACTCAACCTCAACGGAAGGT
AAGACGCGATTGCTTAAGATGTCGTTCCTCATCGTGAGTTACTACTGAAAGATGTGGTTTTGCAAGTG
CCATCGCATGCGCTAAGTTTTTGATGTTAGATGTCGGAGCCAAAATTTACATCGTTTTATTCTTTGTC
GGCAGGTCTCTTTTGAACAACGACGACCCGAACGCCTTCCCTTACTGCAAATGTACTTATCGTCAGCG
CCGGAGCCCATACCGCCTGAAGTATGTTGGCGCCGAGAACAACTACAAGGGCAATGACTGGCTGTGCT
ACAGCATTGTCCTTGACACCACGGGCACTGTTTGCCAGACAGTTCCGTTGACGGAGCCTTGCTGCAGC
GCTGACCTGTACAAGATTGAGTTTGACGTCAGTAAGTGGTGTGATCCAGGCACCCTTCTGTTCTTACC

CAGGCATGTCTGTAGGCACCTTGCCTGGAAAGGCAATTTCAATCTTGCGACACCCTGTCTGGCTCTTC
GTAGCTATGAGTTGGCATTCAATTCCTTCCATCGGCCAAGTAGCCCTTGCCTTATCTCTCTATCCAGT
CAACATCCTTGCGGCCTAAAGTAAGCTCATGGTCCCAGGCCTTGTGGCCCACTGTTACACAGCCTTAC
TAACTGACGCACGTTGTTCCCTTGCCATCCTTTTCCATGGCGCACGTTACAGAACCCTCCTGCAAGGG
CACTGTTACACGTGCTATGGTGTTCAAGGGCATTGACAGGACTGTTGGCGGCGTACGTGTGTTGGAGT
CCATCAGCACCGTCGGCATTGATGACGTCACCGGTGTTCCGGGCGCGGCCATCCTTCGCATCGTGAAG
GACCTTGCCCTCCCGTACAGCGTCGTGGCTTCCTTCTTGCCTAGTAAGTTGGGACTAAACTCAAATTT
TCGTTCTTGCCCAGCAGCTTGATGATTTTCTTCGTGGGGGAAGCGTCACGGTTTGCGATCGATGCCGA
TGTATAAAATGTTTTTTTTGTCTATCTCGCTTTTTAGCCTACGCTTTCTTAATCAGTTTTTTTGGGTC
TTCAGTGTTGTTTTGTGGTCCGTCCCGGCATGTGGACGTTCGTGCCGAAGTTATCTTACACCGAGGGA
TGTGAGGGATGTAAGAAAGGATGTTTGTACCAGACGAATCGTCCACTTCCCATCCAGAGCGCATACAG
CTGCTGCTTTGAATAGTTGCCCATTGCGTTTTGGCGGTTTGCCCATTTTCCAAGGATGCTATTGTCAA
TCGCGTCGGACAAGTTAAGTCTTTTGCACCATAACGAACTTTCCAGCCGTTTAGATGTCAAATAGATA
AAGAAGAATCCTCTTTACGGCGAAGCTCTCACGCCAAAACAAACGTTTTCACTTGAAAGACATTTTTC
TATGATTGCACTTTCCTGGACGGCTGCTCTATGTCTTCCTGTCCCTGCTTGTTCCCTAATCCCTACCC
TACCTGCCTCCCTACCTGCCTCCCTACCTGCCTCCCAACCTCCCTCCCTACCTGCCTCCCTCCCTGCC
CATATCCTTGCAGATGGCTTGCCGGTCTGCATCAACAGGGTGCCCGGCTCGTGCACGTTCCCGGAACT
GTTCATGGACGTTAACGGCACTGCCTCGTACTCGGTCTTCAACTCTGATAAGGATTGCTGCCCAACCG
GCCTTTCCGGCCCCAATGTCAATCCAATTGGCCCGGCACCCAACAACAGCCCGTTGCCACCAAGCCCG
CAGCCAACAGCAAGCTCGCGGCCACCAAGCCCGCCGCCCAGCCC**GCGGCCGC**CAAGCCCGCCGCCTCC ***Not*I**
CTCG**CCCAGCCCGCCGCCCCCACCGCCGCCGCCCCCTCCACCCCCACCGCCGCCCCCCCCAAGCCCCC**
**CACCGCCGCCACCGCCTCCCCCACCTCCTCCTCCACCACCACCGCCACCCTCTCCCTCCCCGCCACGC** **previous sequence gap**
**AAGCCACCGTCCCCATCCCCACCTGTTCCCCCACCGCCATCACCACCGAGCGTCTTGCCCGCTGCTAC**
**CGGCTTCCCCTTCTGCGAGTGAGTGGCAACGGCTTCCCTTCTCTCTCCACTTTTAAACGAACGTTCAA**
**ACTG**TCAAAACGCTACACCAGTACGAAGGATACACAACTGCCGTATCATGGGCAAAGTATTTGTCTTC
AGGTCCATATGTGGTTTCGATACGTCGCTTAGTGCTTGACTATGCTATGCATGTTTAGCTGCGCAGTG
TCGTGCTGTGCTACATTCAGTCATCCAGCGCTGCACAGTGTTGTGCATGATTGACAAAAATACCCCCT
CGGCCATGCGCACCTTGCCCCGTCCAGCCCACGTCTACTTCCTTCAGATGTTCTGTGTACACCAACCC
CCTGCTCGATCACAGAAATATCACAGCACGAACTGGACTGTTCCTTGCTTTCAGCATGTGCGCGCGCG
CGCCTGTGTGTGCATATGTGTATATGTGTGTCGAAATGTGGCGGCAATATCGCTTTTTCTCTATTCCG
AGGGAACAGCATCTGCCAAGGAGCACCCCTGTGTCTCATTTTCGCAAGTGTCCTTGTAACCCGTCCCA
ACCCAAACCCACTCCGCGTCCCCTCATGGACCCCCCCCACGCGCCATGGGCCCCTGGCCACATCCTG
ATACAACCAAAGCTTGCCCTCTTGAGGTGCTATATGACCTGCTTTTCACTGACCCCCTACAACCCCAA
AACTCACATCCTACGCGCCCTCCTCTTTTTCCCCCGCGCCCAGGTGCGTGTCCCGGTCCCCTTCCAGC
TACCCATGGCGCGTGACCGTCGCCAACGTTTCCGCGGTCACCATCTCTGGCGGCGCTGGCGAGCGCGT
GTGCCTGAAGATTTCGGTCGACAAC**GCGGCCGC**TGCCACCTGCAACAACGGCCTGGGCGGGTGCTGCT ***Not*I**
CGGATGGACTGGAGAAGGTC**GAGCTC**TTTGCTAGTGAGTACCTCAGCGTTGCGGCGGCAATTTGTGTG ***Sac*I**
AATGTGTGTTTGTGTGTGTGTGGGGGGGGGAATGGGGGGTGGGGGTGTAATGGCGGCGCGGGAGATT
GGGATCACGGGACTGACACTACCATCAGATGGCTTTGGGGGAGAGGATGGGATTTTCGGGTGAACTGG
AACAAGGATGTGTGTGTGTGTGTATGTGTGTATGTGTGTTGTTCATGCTGCTCTAGCAGCTGCGGAAA
CAGCTCCTGGGTGATGGGTGGCTTCCTAGGCACTACAGCTACGGGCTATGGCGAGCCCGTCATAGCAG
CCTGCTGGGAAGCCGATGTGGTGCGTAACGTTGGTCGCTTAGGAGGGGTCGTGGAGGGGTCGTGGAGG
GGTCGTGGAGGGGTCGTGGAAGTATCGATGTTGCGTTTGCGGTGACCCAGCGTGAAGAATAACAAATG
CCAACAGTGTCGGAGTGTTATCAGGTGCAAAGTCCTCCGCAGTGCTGATGCCTCCTCTTCAGCAGCAG
CCTGCCCTACGCGATTACACGGTGGTTGAAGTGGTTGCTGGTTGGCGCCGCCAGCATTATATTTTAAC
CACGTCAGGGCCTGATGCGCCGTGGGTTTAGGAAGCAGCTTCTCGCCTGAAGGGAGGGGGAGACGGGT
TCTGGGTTGGTTTGGTTCTGGTGCCCGCTAGGGTGAGAATTGCTGAAGGGCTGACGTGTACCAGGTCA
TATGTACCCTAGTGGCGCGAAGTAAACAGACGAGCTAGGGAACATAACAATAACAACAGCAAATACAA
TAAGCTTTAAAACGATCTATTGATATGCGCTGCATGTGCGTGCGTATGTATGTGTGTATACGGCGATG
CGAGTACGGCATCTTTCCATACTCTGGTCTTTAATCTCACCCCTGAAACACACCACCTTCGTTTGCGC
GTGCATCCATGCGTGTGTGGTGTGGGCTGGCGTGGCCTAGTAAGTGGAAATGATTTCAAAGCCAAAAC
AAGCTACCGGGATAGCATCAGTCTCTTCAATCATTCACCCGGTCTCCGCGTGTGGCGCTTGGGGGTAT
GTTCCCACTGCCGCCACTGTCACCTGCTGAGCTGTCGGCATGTCGTAATAACGCCTCTGCACGCCAGC

CCGTGGTATCGTTTTCCGGGATCCCGCTGTCGGGTCTAAGAATGGCTTCCGTTGCTGGCGCTGCTCGC *Bam*HI
TCGAACGCGAGACCAAGTTAACTTTTACTCCTGGAGCAGAAGGTTTAGTAAAACAAGCTATTGCTGAA
TATTTAGAAGAATTCAAATCAAGTGCTAAAGCTGCTTAATTAGTAATAATTTATATAATTTGGTGTCC *Eco*RI
ACAACATACATAGCTGCCGGTTGCAACCACATCTACACCCACATATGTCTGTGCGCGTGCGTGTTGCA
CCTTGGGCTGCTTATGTGCTACCCTACGAATGTATTAACCTAGAGCCCCATTTACATGTGCTCAACCA
TGCCTACCAACACAACACCAACACATTTCCCCTTCGCACCCGTTCTCTGTTCGAACCCCACAGACGGC
AAGTGCAAGGGCTCCATTTTGCCGTTCACTCTGTCCAACACCGCGGAGATTAGGTCGTCATTCTCCTG
GGACTCCACTCGCCCAGTCCTCAAATTCACCCGCCTGGGCCTCACCTATGCTCAAGGTGTCGCCGGCG
GCAGCCTCTGCTTCAACATCAAGGGTGCCGGCTGTACCAAGTTTGC**AGATCT**CTGTCCTGGCCGCGGC ***Bgl*II**
TGCACCGTCGCGGTATTCAACAACCCCGACAACACATGTTGCCCGCGGGTCGGCACCATCGCG**TAA**AG **stop**
CGTTTCGCATGACGATGCAAAAAAAAGGGTGGTGCAGCGCAACACCCCCCCGTGGGTGTGTGGGTGTG
TGTGCCCGCGCAAGAAGATTGTGCGTGAGTGGATGTGCGCGGATGTGGGGGGGAAAACCGCCAGTCCC
TTTCTTATATGCTGCTACACATGACTGAGGTTGCTGCCGGGGAGGCGGAGGAGGCTTGTGGGTGTGGC
GTCGGCGTCGATGCCAAGTCACCGCTGTGCCACTGCTGCGGCGGGCTTGGATAGCAGCAGCAGCGGCG
CAGCAGCAGCAGCATCCGCAGCATCAGCAGCATAGGGACGCATGCATGCCTTTAGCCGGAATGGGAGG
GCTGCGTGCTGCTTTTGCAACCGCAGGGGTGAAAGTAGGTAGTCCTGCATAAATGATTAAGCACTGGT
CAAATGTCGCTGAGATGAACATAACACGATTATATATGATATGATGCGCGACATGATTGTTGGATGCG
GCCAGTACTTTTTTCCGCTGCGCAGGTGTCAGTGTTGCACTTGGGTTTGATCCAGTTACGGGCCTCTC
GGCGTCTACCAAACGCAAATTGTCGTTAATTGATATTCTCTTGCTTTTTGCGCATCTCGTCAAGTGAG
GGCAGCGTCCCCCTCCTGTGCTGCTGCACTTGACGTGGCTGTTGGAATGTTCCGTGCATGGCCGCTGG
CCCGAACCATTTGTAGTTGAGTGACTACTGCGCTAACATTCAGTTATTTCGTAATAATCACCATCATA
ATTATCATCATCGCTACCTGCCTATGGCCGGGAAGTTGAGCAGGTTTTAACGTTTTACGTTCAATCCC
GATGGCCCACGTCACGTGTAGCAGGCCTTTGCGTTCCCGCTCTCTTGTAACTTATTTCTCATA**GATAT** ***Eco*RV**
**C**AAATTTGGCTCATCTTTGCAGTGGAGTATTCCTACAAATAGAGGTGCATCTGCCAGATTTGGCAAAT
AGAGGTGCATCTGCCAGATTTGGCCGGTGGGGGACCTTATTAACCGTCGTTAACCA**GGATCC** ***Bam*HI**

The sequence depicted here corresponds to the 8.8-kb genomic fragment utilized in plasmid pSSG185-YFP. The *ssg185* gene is located on scaffold 2 (nucleotides 4191410 to 4198020) of the *V. carteri* genome version 2.1 (Prochnik et al., 2010) in Phytozome 12.1.6 (Goodstein et al., 2012) and has the gene ID Vocar.0002s0564. The start codon is at nucleotide position 4191698 to 4191700 on the forward strand. The gene structure is indicated as follows: Coding sequences are shown with blue background, UTRs with green background and the promoter region with grey background. Start and stop codons are highlighted (violet font). A previous sequence gap, which we closed, is also indicated (blue font). The 5' UTR is 153 bp in length, while there is a quite long 3' UTR of 891 bp. The restriction sites that are shown in Supplemental Figure S5A and B are marked (bold, underlined).

## References


**Goodstein, D. M., Shu, S., Howson, R., Neupane, R., Hayes, R. D., Fazo, J., Mitros, T., Dirks, W., Hellsten, U., Putnam, N. et al.** (2012) Phytozome: a comparative platform for green plant genomics. *Nucleic Acids Res.* **40**, D1178-1186.

**Prochnik, S. E., Umen, J., Nedelcu, A. M., Hallmann, A., Miller, S. M., Nishii, I., Ferris, P., Kuo, A., Mitros, T., Fritz-Laylin, L. K. et al.** (2010) Genomic analysis of organismal complexity in the multicellular green alga *Volvox carteri*. *Science* **329**, 223-226.
