# Supplementary material for: Cell Type-Specific Pherophorins of Volvox carteri Reveal Interplay of Both Cell Types in ECM Biosynthesis
Source: Cells. 2022 Dec 29;12(1):134. doi: 10.3390/cells12010134 (PMC9818292; doi:10.3390/cells12010134)
Supplement: Supplementary file 1 [file cells-12-00134-s001.zip › Supplemental Figure S8.pdf]

Supplemental Figure S8

Quantification of PhG:YFP fluorescence intensity in the gonidial vesicle before, during and after embryogenesis

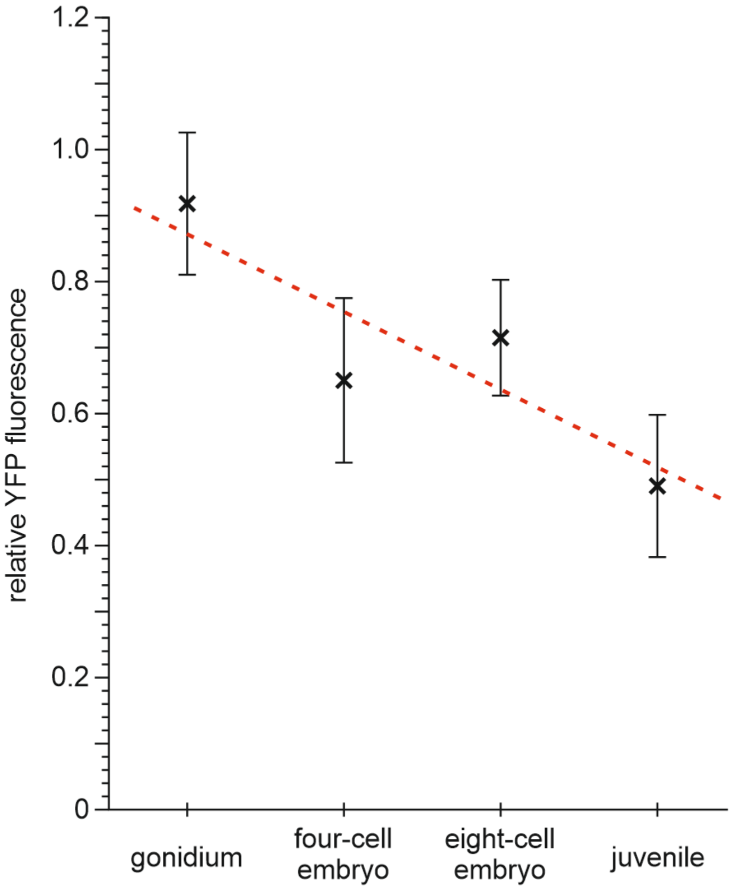

In *V. carteri* transformants expressing the chimeric *phG:yfp* gene under control of the endogenous *phG*-promoter, fluorescence intensity of PhG:YFP was quantified in the gonidial vesicle of gonidia, four-cell embryos, eight-cell embryos, and juveniles just before hatching. Since parameters such as digital gain, laser intensity and focus settings vary between different fluorescence images, normalization was required. Therefore, for each developmental stage, ten regions of interest (ROIs), each with 150 pixels, were analyzed for the highest fluorescence signal in the YFP channel (detected at 520–550 nm) and in the chlorophyll channel (detected at 650–700 nm). For each ROI, these two intensities were normalized to the digital gain used in taking the corresponding CLSM image. The YFP signal was then also normalized to the chlorophyll signal of the same ROI to compensate for differences in laser intensity and focus settings. The data shown are means and standard deviations of the ten measurements for each of the four developmental stages. A linear approximation (dashed red line) illustrates the trend of PhG:YFP fluorescence intensity as development of the offspring progresses.
