# Supplementary material for: Cell Type-Specific Pherophorins of Volvox carteri Reveal Interplay of Both Cell Types in ECM Biosynthesis
Source: Cells. 2022 Dec 29;12(1):134. doi: 10.3390/cells12010134 (PMC9818292; doi:10.3390/cells12010134)
Supplement: Supplementary file 1 [file cells-12-00134-s001.zip › Supplemental Figure S1.pdf]

Supplemental Figure S1

Sequence of the chimeric gene *phG:yfp* as it is present in the vector pPhG-YFP

GGTACCTGAACATGCTTTTAGGACTTTCAATCTGTGCACCCGAACACACAAGTTACAATCAACGCTGT ***Kpn*I**
AATGCGGGTAAGTTAATAATTAATGCATTTGTCAAGCAATCGGTGTTTGACATCCGTGATAAAGTCTC
TTCAGAATCTGCAGCCCGAGTGTTCAATTTGTTCATTAAAACTTGTGAAAAGTGTATGAATGTGTCTA
TTTGGCATGTACTTCTGTGTTAATGATACAGCCGGTGATACGCGTTTGATACCACTCGTTATCCGGAA
CAATCGACCCGTATCGACATGATGTAGATGTCCTGGGATCGCTGATGGATTCCCAAAAATTCTTCTGT
TGCTTATCTTCAGAACAAAGTACTTCAATCTTCTATGCTAGAAATACCAGTGCTCAACGAATGCTCCT
GCCTGCCGCCACCACATCTCGCTGTATGTGTCGCAAAGGCGGGATCCGCATATTGAACAACACCTTTC
TCAATTGACTCCAAGCACCAATGTTGCCAATATATTTATTCAGACATTGCGATATATACTCCAAGGCA
AGGAAACGAACGGCATCAATGTCAAGTGCGCTAATGCACGCACGGGTCGCGAGCGCTACAATGCTGAC
GAGCCCATACTTCAATCAGGAGTAAGCGACGAAAGTGAGGAATATCGACTAAGCGATGACAGTGAGAA
ATCGTTAATTGTGCATAACAAGCGTGATTCCCGCTTGAAGGGTTGTAGCTGCATCTAGCTTTTTGCCC
GTGGCAGCAGATGGCAAATATCTACCATCGGCTGTCAAGTAAATACGGTTTTGTCGGGGATTTAAAGT
AACGAAAGGTTCAAAGGAACGGAAGGAGCTACACATTGGGCTGCACGCGACTGATTTCAACCAGCTCC
ATTGTCGTACTGTACGTTCAAAGTAGTCTGAAGTAAAGCTGACTATAAATACGCGGTTGCCAATTATA
C**GTCGAC**ATACTAATTGGGCACAT**AGATCT**CCTAC**ATG**CGAACACCAATGAGGAATATACTGCTGGCT ***Sal*I, *Bgl*II, start**
GCTCTTTGCGTAGTCGCCGTATTCTCGTAAGTGTCGTTGCTTACCTTGCTAGGGCCAGCTGCAGGCTT
CGATGTACAAGCATGTAGGGTTCGCAGGCTTATTCTTAAACGTTTACCTACCTTCTGAGCCATGAGGA
GTCGTAGCTTTAATAGCCATGCTGCAATCCGTTTGGCTTACGTCGCTTTGGCGGGCACGGGCGCCAGT
ATCACCCCGTGTCGTTGATCGCTGCGGCTCCCCTCATTTTGCGAAATTCACCATCATATTTGTAAATG
ATTGATGACTTGCTGAATCTCTTGCCACCTTGCGCTTATTTTCCAATCTGCAGGGCGTCGCACGCGCT
GAGTAGTCCGCTGTCGCCCCCGCCGACAAGATACCAGGCATTTCCTTACTGCGCAAAATGCGTCGCTT
TGGAGCCTATATACCGTCTAAAGCCTGATTTTGCCGGATTTGGAAATCGCACCTTCTGCTTCACGCTG
GAAGTGAATCCTCTCAATGCCGACTTCATGTGCAAGAATGCATTAATCTACAAGATGGATGTGAGTAT
ATATCAATTGAACATGTGTTGATGTAGCGCAACTAGCACTTCGGGCGGTTCTCGAAATCTAGCAGCCT
TTCGCGTTACGGTTGTCGAACGTTAAAGCTTTAGTCAGTCACGAGGGCGCGCAACTGTGTGTCACTCC
ATTCGAGCAGATTTACCTTGCCTGGTTGTCTTCATGACATAGAACATAAGAAGGGTTATCTACTTCCA
TTTTTATTATCGTTAACAGCGTTCCCCGGCGAACCCGCAGATGCTTCTCAACAAAGGGTGCGGAAGGG
ATGTTAACGTCAAGGCTTTCGTAAATGGCGTCTTCACAAGGAGCTTCACAGAATCTCCACGTTCTGCC
GCAAATGGGACTGTGATATTGAAACTGAACGATCTGACCGCGCGACACCTGGGTATCAACAGCAATGG
CGCCAGGATTTGCCTTACTATATTGAATGGGGGCAAGGGCAACTGCACCACTTTGAGGTCGCTCTGTG
CAGACCCTAAGACCACCAACTTCAGAAAGGATAACAACGGCACTTACAGAAATACAAATTCCCAAGAG
TGCATGGTTGCGCTATTCGCCGGGATCGACAGTGGCTCCGACCGTACCTGCTGCGACCCCGGTTCTCC
CCCATCTCCAACGCCGCCCCCGCCTCCTCCAGGGAATTGCACGGAGGAGAAGGTCTGTGTCTATCTGA
CCTTTGGGTTCCGCGAACCG**GACGTC**GCCTTCCCCAACGGCACCTGCAATAATTTGGCCAGCCAGATA ***Aat*II**
CAGTTCGACCTCCTCAACTCAAGCGCATCCTACATCTTGCAGCCCCCGGTAGTTCCACAGATCACTTG
CACAGCAGACTTTATCAAAGTTTGCTTAAATTTGGACTTGCACGCTAACGCTACAGAGCTGCATTCCG
TGGTTGGTGAAAATGCCGGTCCCTGGCTGAATTACGCTCACAACTCCACGTGTGCGGCAAGTCGCTCC
AACTACAGCCTCACAATTACGGTCGGAGGGGATGGCGACTG**GTCGAC**TGGCAACGTCTCGTTCCGTTG ***Sal*I**
TTTCAATGCGTCAACTGAAACCAAGTGCCAAGGGCCCTCGAAGAAATCATGCAAGTGAGTCGAGCAAA
CCTGGAGGAGGGGGACAAGTATTGCGTGGCGGTGGGGAGTAAACATGTCTGGCGTTGATCTAATGTGG
GATTGCATGCTCGACGCGGATGCATGCACGCATCCACGTACGTGTCAGCAGCTACATCTTCCTGAGCT
ACTGGTTTGAATTGGATTTTAGCAGCTTTGTGCCCTCACATGATTATCCTTCCAGCTGTATCCCTCTG
CTCTCGCCACCTATCCCTTTCCCTCCTTTCCTCTCCTTCCTCCGCAGCTGCAATATGGCGAGGAACAT
CACCCCCTTCGCCGCCTTGCCGACTATGAGCGGTCCCTTCACGGGCCGCAAGAACACGAAGCTGTACT
GCTTCAACATCACCGTCGTGAAGCCCATCGAGCCAAATGTGGGTCGCAGCGCTCGCTGGCGCCGCCGT
ACACCGCATGATAGCATGTGCATGTCCTGGTTGATGTATGTATGTATGTATGTGGCCATCTACTGGCT
GCGACACGAGCACAGTAGTAGTGCCACATTGACGGCTACATCGATTACGCCTGTACAGATGTCTAAAT
GGTGCATGCGGCCACATCTTCCTCCAGTAGCACTCCATTACGGTTCCCTTGCTATCCAATCCTATCCC
TCGCATATCCCCCCTTTACAGAGCACCTGCGGTAAAAGCAATGTGCTCGATAAGGTCATCATCTGGGC
CAACGACACCAGTTTTTACCGCAGCAGCATCAAGTCCATCGCTCTCTACGCTGCGGGCGACACCACCG
CGAAGTATGTCCCGCCGAGCTGGAACTCGGATAGACAGGAGGTGAAGGCAACCAAGATTGGCTGGACT

AAGGATAAGGCGAACGGCGGCGCCATCTGCCTGGAGCTGGACCAAAACGTCAACCTGGCTGACTTTTG
CATATGGCGGGGTCCCAAGCAGAAGAACTCTTGCATGTAAGGGCCTGGGGATTTATTTGGGGGTTGCC
TGCATGCTTGTCATGCAGTGTGCATGCATGGCGGGATGTGAGTGTGCGCGAACTAGGCGGCACTCGGT
CGCCATCCCGGCTGCAGAAATGCAGGCATGCACCTAGCTAGACTATTTACGCGTGCTTCCTTGCACAT
GGTTGAAGAGCTCTCCAATGGCACCCTAGCCTTGCACTAGCTTAGCTTTTCTACTGCTTAGAGGCGCC
TCTGGGCCGAGCGTCCTGTCTACCTACCTTATTGTGCGTCCCCCATGCCCATGCCCATCTTGCTGAAT
GCAGGGTTTTACTTTTCGATACATCCTATAGATGCTGCCCGATGTACCGGCCGATGGCGCTTACTACG
ACTAGTTCTAGAGGCGGAGGCGGTGGCATGAGCAAGGGCGAGGAGCTGTTCACCGGCGTGGTGCCCAT ***Spe*I, *Xba*I,** gly5
CCTGGTGGAGCTGGACGGCGACGTGAACGGCCACAAGTTCAGCGTGAGCGGCGAGGGCGAGGGCGACG
CCACCTACGGCAAGCTGACCCTGAAGCTGATCTGCACCACCGGCAAGCTGCCCGTGCCCTGGCCCACC
CTGGTGACCACCCTGGGCTACGGCCTGCAGTGCTTCGCCCGCTACCCCGACCACATGAAGCAGCACGA
CTTCTTCAAGAGCGCCATGCCCGAGGGCTACGTGCAGGAGCGCACCATCTTCTTCAAGGACGACGGTA
ACTACAAGACCCGCGCCGAGGTGAAGTTCGAGGGCGACACCCTGGTGAACCGCATCGAGCTGAAGGGC *yfp*
ATCGACTTCAAGGAGGACGGCAACATCCTGGGCCACAAGCTGGAGTACAACTACAACAGCCACAACGT
GTACATCACCGCCGACAAGCAGAAGAACGGCATCAAGGCCAACTTCAAGATCCGCCACAACATCGAGG
ACGGCGGCGTGCAGCTGGCCGACCACTACCAGCAGAACACCCCCATCGGCGACGGCCCCGTGCTGCTG
CCCGACAACCACTACCTGAGCTACCAGAGCAAGCTGAGCAAGGACCCCAACGAGAAGCGCGACCACAT
GGTGCTGCTGGAGTTCGTGACCGCCGCCGGCATCACCCTGGGCATGGACGAGCTGTACAAG**GATATCG** ***Eco*RV**, ***Eco*RI**,
AATTCTGGAGCCACCCGCAGTTCGAGAAG**TAA****TCTAGA**TAGATGCGTGCCGTCACAGCTGGCTAGTCT strep tag, **stop**,
TGTGCAGAGCATGCACGTTGGATCACTGACCTGGTGAGGCGAGGAAGCCCCATCTAAGGTTGTGCGAT ***Xba*I**
TGGGCGATTTGTGTGTTGCGTGGTAGGATGTAGAATATTTGGTTTGCGAATGTGCTTTGGATAAAATC
CATGTTGGTAGTTCCTAAACTTGTGCACGATGCTGCCCGTGAACAGGGCAGGTAAGGTTGTGTGTTTG
GAAGTGTTTGGAATTACATGGAGGTTATCTCTAAGTTTCAGTGATTAACAGTGCTTAAACAGTCATGT
CTGGTTTGCGGCTGTTTGCGTGTTCCTGCCTGTTCTTACCGGCCGTGTTGCCTGCTGGACCTGCCGCT
GCTGCGCAGCCTACCCGTGCGATTTCGCTAGGAAGAATTTCATTTCGTTGTGCAGGTCGCCTTTCTTA
CTTTTTCAATTCTGTCTGTCAGAGGTGCTTGCTTCCTTGCAGCAGGTGGAACTGATCACCTCGCCGCC
GTTTCGATCATTTAAATACCCCCTACGTCGCGCAATGATGTGGGGTATCGTAGGCTGCTTTATGCAGC
CTCGTTCGCTTATTTGATCGCACCTGCGATTGTAGCAGCTTCATTCATTAGCTGAATCAAGTGAGCGA
GCACGCTCGGTCGGTCAGGTTTTATAACATGCTGCTTGCTGCTCAAGCTTTTGCTAATATTGTATAGC
GCAGGTACGCTTTGCTGATTGCTTCTGGGGTCGCCTAGTTCGGGGCTACTGCTTGCATGCTGTGTGTG
GTGAGATAGCAGGCTGGCTGCCATTACTGCGGAGTACGCAGCTCTTCAGCCATATATTCATAGTTAGG
ACGAATTGATTAGCCAGC**CCATGG**CGATGTCAGTTCAGTTCAATGTCCATTTTCATACGCGCCTTGCG ***Nco*I**
TTTTGTTTATGGCTGTCAGTCTGCGTGATCACCTTATGTTAGATCGATGCATCCTGCAATCGTTTTGT
GCGTTGTAACACTGCGAGCGCGACAGGCAGAGTGTTAGGCGTATTGGCGAGAAATGTCCTGTTCGGTT
AGGGCGGTTTGGTTAGGGCAGTTTCTAAAGATCCTCTGCCGGCTGATGGAAATATCGGTGTTTATGTG
TAGGAAATTTCTCCCGCCCGCCATCCATCCCCAACATTACCGTGTAACATAATCCAACAATCCTTGGC
CCATCCTATCCTATCCAAGCAGGTCTTCAAGCATACTTTTTCATCTCCTTCACACTGCAGCCGGCAGC
AGGCATGATTCCTGCGAAC**GCGGCCGC** ***Not*I**

The vector pPhG-YFP carries a 5.3-kb fragment of *V. carteri* genomic DNA containing the *phG* gene (*phG*) including its 5 introns, a short linker sequence, which codes for 5 glycines (Gly5), a strep tag and the coding sequence of *yfp* (mVenus). The chimeric gene is controlled by the original *phG* 5' and 3' flanking sequences, including the promotor region. The corresponding gene entry in the current *Volvox carteri* genome version 2.1 (Prochnik et al., 2010) in Phytozome 12.1.6 (Goodstein et al., 2012) is Vocar.0001s0298, the former common name is *phV47* (von der Heyde and Hallmann, 2020). The *yfp* (mVenus) gene has been codon-adapted for *C. reinhardtii* (Lauersen et al., 2015) but also works well in *V. carteri* (Tian et al., 2018; von der Heyde and Hallmann, 2020). Artificial *Xba*I, *Not*I, *Bgl*II and *Spe*I sites have been introduced to facilitate cloning. The background colors indicate the following sequence features: grey, *phG* promoter region, green, 5' and 3' UTRs of *phG*; blue, coding sequences of *phG*; turquoise, sequence coding for 5 glycines (Gly5); orange, sequence coding for strep tag; yellow, coding sequence of *yfp* (mVenus). Start and stop codons are highlighted (violet font). Relevant restriction sites are marked (bold, underlined).

References

Goodstein, D. M., Shu, S., Howson, R., Neupane, R., Hayes, R. D., Fazo, J., Mitros, T., Dirks, W., Hellsten, U., Putnam, N. et al. (2012) Phytozome: a comparative platform for green plant genomics. *Nucleic Acids Res.* **40**, D1178-1186.

Lauersen, K. J., Kruse, O. and Mussgnug, J. H. (2015) Targeted expression of nuclear transgenes in *Chlamydomonas reinhardtii* with a versatile, modular vector toolkit. *Appl. Microbiol. Biotechnol.* **99**, 3491-3503.

Prochnik, S. E., Umen, J., Nedelcu, A. M., Hallmann, A., Miller, S. M., Nishii, I., Ferris, P., Kuo, A., Mitros, T., Fritz-Laylin, L. K. et al. (2010) Genomic analysis of organismal complexity in the multicellular green alga *Volvox carteri*. *Science* **329**, 223-226.

Tian, Y., Gao, S., von der Heyde, E. L., Hallmann, A. and Nagel, G. (2018) Two-component cyclase opsins of green algae are ATP-dependent and light-inhibited guanylyl cyclases. *BMC Biol* **16**, 144.

von der Heyde, B. and Hallmann, A. (2020) Targeted migration of pherophorin-S indicates extensive extracellular matrix dynamics in *Volvox carteri*. *Plant J.* **6**, 2301–2317.
