# Supplementary material for: Cell Type-Specific Pherophorins of Volvox carteri Reveal Interplay of Both Cell Types in ECM Biosynthesis
Source: Cells. 2022 Dec 29;12(1):134. doi: 10.3390/cells12010134 (PMC9818292; doi:10.3390/cells12010134)
Supplement: Supplementary file 1 [file cells-12-00134-s001.zip › Supplemental Figure S2.pdf]

Supplemental Figure S2

Sequence of the chimeric gene *ssg:yfp* as it is present in the vector pSSG-YFP

GAATTCACATGGCGAAGTATAAGCATTGGATGGGGCTGGCGGCCGAGCAAGCGGGCAGGAAGGAGGTG ***Eco*RI**
CTCCCGCACATCACAATGTTTATCCCACGTTCGGCGAATTGCAAGCTTATGCAGTTTAGGCTTTGCTG
TTGGGCACTGGCGGCTAACAGAGCGCGTAAGGGACGGCTTTATATACCTAGGGAGGAGCGAGTATGCC
CCTGCTGCGGCACTGATAGCCACATCGAGGATGAGAAACACATTTTGCTGGACTGTCCGGTTTACGAC
GGAATCAGATCTGATATGGGCTTTCCACGCTTCGAACATATGAAGGACGCTATGAAGTGGGAACCTGG
ACGCCTGGCAAGAATGCTGGAACGTATATGGTCTCACCGCAACTCTATCGTTCCCTTTGGTCGTTAGT
ATACTTCATTACTTGTTTCGTTGGAAGTGCGAGGTAGGATGGTGCTTATAGGCGATTTGGCAGCCCTT
GAGCCCTCGGCTCGTACTAGGGTGGTTTGCAGGTACGGGGAACGCACCCACGACCATGTAACAACAAC
AACAATACATACGTGCCGTCTTCCCTGAAGCATATTTTCCCCCACGATGAATCGAATTATGACAAAGT
CAATATCAGGTGAACCATACGAACGGCGCCTCTTCGTGATGGGGCGACGCAGTGACTTGATCCGCCGA
GGGAAACGCCACCCCATAGAACGCCTGCAGAAGCTACAGCACGGCGGGCCGTAAAATGGAGTGTGGTT
TTGGTCCCCGACAACGATTACATTGACATGCAAGCAGCGGGTTGATTGCAAAGCTCAAGTTCATGCGA
CATCATCATTTGTATTTGACGCACATCGTGGCCGTTTTCCCCTCCTGAGGAATGAGAACGATCAGGAA
GCAAGCACGAAACTGACGCTGACTGTTGTTGCTTGCGGGTGCGAGTCAGCAACACATGCAACCCCAGT
TCAAGGGTCCAATTATGAATGCGGGGATGGGGTGGTAGGCGCGGCAGCGAGTATGCGCGTATCGGCGC
CCGGCTGGGCATCAGCCTGTTTGAGACGCCGTGCCATGCCGCTCCGCGCGGGTGCTTCGTTGGTCCAT
ACGTCTCGACTGGAATGTGTGTAGTCACAACTTCTGCCACATTCGATCTCCTTGGTCTTTGTAACACT
TTTGGGCGAGCAAATTTTAATCACTTTTTGTTGCCAAAGCGAAGGTATGGACTGGGCTTTCTAAGTCC
CGTTTCAAAAGCACATCTTTCGGAAGAAAAGGATACGAAAAGTTTTCATTTACGGCCGCATGTGCTAT
GTGGGTAGATTTTGCAGTGGCAGTTCCGCAATTCGACAACATGCCTTAGAAAATAAAATTAAGCTCCA
AGCCCAGGGCCGAAATAGAATACTGTCTATGTCTCGTGCAATCACCGGCAATTGAAGGAAGCAAGAAG
CTATTGAAATTGACCCTGAGATTCCTTTGAACGACTTATTCTCGATTGTTACGGCAGTCGCCCACGTG
AAACTGCCGCCGTTCATCAGCAGTCAGGGCAAGCTAGCGTGAAAAACAGTAACGCGCCGTATCAGTAG
TACATGTTTGAACGGCGTTTTGCTTGGATAAAATTATGAAGTTTTCTTTTGGCAAGCAGACCTTTGGT
GCATCACCCTTCTTTATAAGCAATTCATCGCTTGCAACGCACATACTAGCCAACTTCTTTTAGCGATA
TACTTATTGGTATTGTTCTAGTACTTTCTATGAACGTCGACTGTGTCCAATTGTGTTCAACTAGGATT
AAGCGTGCCACGTCAGCAGATAGACTGCTAGTACTGCTGTTCTCCCGTGTATGACTACGGTACTCTGA
AAAGGCGTTAACGCCTCATATTAACGTAAAGGTAACGTA**GATATC**AGTTGCTAAAATTACCTAGCATT ***Eco*RV**
AGATTATGTTGCAAAACGGATCAATACGTATCGGATAGGGATGTGGCGAAATTGCACGACGCTCGGAA
TGAAAGGTAGAATGCGTAAATGTTCTTACCTTTCCCTAGACTGCGAAGAAACAAGCAAATGATGACTT
GATCTATTTATTTTATCCAAAGGCGCCAATATCCGACGTCACATCAACCGAGCACTCCATCCAAGTTA
GCCATTGAAGCAGGAGCAGCAGCACAACAACGTAATCACCTATGAGGATATAAGTCTCATCATATTTT
AAACTATATTATACTGCACTCAGGATGACTGCCGGAAGGGAAGTGAACCGCCGGCAATCTTTACGAAG
TAGCTGGCCAAATCCATAAGCCTCATGAAATTATTAAATCAAATTAACTCTGATTAAGTTTAACTTCA
TCGCGGATGTCACCGCGTACGTTGGACTTGTCGTTACCCATCAGCATTGCTTACTGTTAACTTGGCGG
AACGTTACAATCACATCCCAGGAGGCCACCATCCTCAATATACTCTATTTTATGGTGTTTTAAGCACA
TTAGTTGGCACGCTCGCCTTCTTTCCCGCGCTCGCGCCCACCGCTCTGTACACCGTCTGGACATGCCG
TACATTATTCGCCAGGGAGGGCGCGTTGCAAGCCGTCATAGAAGGATCGGGTTCTGCGATTGCATAAT
CGTAGCTGCACCCTTACAGGCTGAAGAGTTACGGCCTCGTGGGTTGCATAATTGGAATCCCTTTTTCA
CGAAAAGGCGTGGAGTGGTCCGTTATTCCATTTCTGTCACGA**CTCGAG**ACTTGGCTACGTAAGTCTGC ***Xho*I**
ATGTGATGGATTCACGCGACATCGCTAGATTGTTTGATGTTGCATAATTGGCCAACATCTCAGCATTT
TAGCTGGAGAGGGTAGCTGGCAGACGCTTATCATGTCGCGGTTCCTTCCGTGCGCAGTGGTGCTCACC
GGGACCAATTTTATACGCAAAG**ATG**TCTAAGCTGCTGCTCGTGGCGCTTTTTGGCGCCATAGCAGTGG **start**
TTGCTACGAGTGCTGGTGAGTGCGCTGCCTCCTTCGACCCTTACGATGCAACGTAAATTTTCGCTACC
TGAGTTAACCTGCTTCTTTCTGCCTATTCTTTTGCTTATAACAGAGGTACTCAACCTCAACGGAAGGT
AAGACGCGATTGCTTAAGATGTCGTTCCTCATCGTGAGTTACTACTGAAAGATGTGGTTTTGCAAGTG
CCATCGCATGCGCTAAGTTTTTGATGTTAGATGTCGGAGCCAAAATTTACATCGTTTTATTCTTTGTC
GGCAGGTCTCTTTTGAACAACGACGACCCGAACGCCTTCCCTTACTGCAAATGTACTTATCGTCAGCG
CCGGAGCCCATACCGCCTGAAGTATGTTGGCGCCGAGAACAACTACAAGGGCAATGACTGGCTGTGCT
ACAGCATTGTCCTTGACACCACGGGCACTGTTTGCCAGACAGTTCCGTTGACGGAGCCTTGCTGCAGC
GCTGACCTGTACAAGATTGAGTTTGACGTCAGTAAGTGGTGTGATCCAGGCACCCTTCTGTTCTTACC

CAGGCATGTCTGTAGGCACCTTGCCTGGAAAGGCAATTTCAATCTTGCGACACCCTGTCTGGCTCTTC
GTAGCTATGAGTTGGCATTCAATTCCTTCCATCGGCCAAGTAGCCCTTGCCTTATCTCTCTATCCAGT
CAACATCCTTGCGGCCTAAAGTAAGCTCATGGTCCCAGGCCTTGTGGCCCACTGTTACACAGCCTTAC
TAACTGACGCACGTTGTTCCCTTGCCATCCTTTTCCATGGCGCACGTTACAGAACCCTCCTGCAAGGG
CACTGTTACACGTGCTATGGTGTTCAAGGGCATTGACAGGACTGTTGGCGGCGTACGTGTGTTGGAGT
CCATCAGCACCGTCGGCATTGATGACGTCACCGGTGTTCCGGGCGCGGCCATCCTTCGCATCGTGAAG
GACCTTGCCCTCCCGTACAGCGTCGTGGCTTCCTTCTTGCCTAGTAAGTTGGGACTAAACTCAAATTT
TCGTTCTTGCCCAGCAGCTTGATGATTTTCTTCGTGGGGGAAGCGTCACGGTTTGCGATCGATGCCGA
TGTATAAAATGTTTTTTTTGTCTATCTCGCTTTTTAGCCTACGCTTTCTTAATCAGTTTTTTTGGGTC
TTCAGTGTTGTTTTGTGGTCCGTCCCGGCATGTGGACGTTCGTGCCGAAGTTATCTTACACCGAGGGA
TGTGAGGGATGTAAGAAAGGATGTTTGTACCAGACGAATCGTCCACTTCCCATCCAGAGCGCATACAG
CTGCTGCTTTGAATAGTTGCCCATTGCGTTTTGGCGGTTTGCCCATTTTCCAAGGATGCTATTGTCAA
TCGCGTCGGACAAGTTAAGTCTTTTGCACCATAACGAACTTTCCAGCCGTTTAGATGTCAAATAGATA
AAGAAGAATCCTCTTTACGGCGAAGCTCTCACGCCAAAACAAACGTTTTCACTTGAAAGACATTTTTC
TATGATTGCACTTTCCTGGACGGCTGCTCTATGTCTTCCTGTCCCTGCTTGTTCCCTAATCCCTACCC
TACCTGCCTCCCTACCTGCCTCCCTACCTGCCTCCCAACCTCCCTCCCTACCTGCCTCCCTCCCTGCC
CATATCCTTGCAGATGGCTTGCCGGTCTGCATCAACAGGGTGCCCGGCTCGTGCACGTTCCCGGAACT
GTTCATGGACGTTAACGGCACTGCCTCGTACTCGGTCTTCAACTCTGATAAGGATTGCTGCCCAACCG
GCCTTTCCGGCCCCAATGTCAATCCAATTGGCCCGGCACCCAACAACAGCCCGTTGCCACCAAGCCCG
CAGCCAACAGCAAGCTCGCGGCCACCAAGCCCGCCGCCCAGCCC**GCGGCCGC**CAAGCCCGCCGCCTCC ***Not*I**
CTCG**CCCAGCCCGCCGCCCCCACCGCCGCCGCCCCCTCCACCCCCACCGCCGCCCCCCCCAAGCCCCC**
CACCGCCGCCACCGCCTCCCCCACCTCCTCCTCCACCACCACCGCCACCCTCTCCCTCCCCGCCACGC **previous sequence gap**
AAGCCACCGTCCCCATCCCCACCTGTTCCCCCACCGCCATCACCACCGAGCGTCTTGCCCGCTGCTAC
CGGCTTCCCCTTCTGCGAGTGAGTGGCAACGGCTTCCCTTCTCTCTCCACTTTTAAACGAACGTTCAA
ACTGTCAAAACGCTACACCAGTACGAAGGATACACAACTGCCGTATCATGGGCAAAGTATTTGTCTTC
AGGTCCATATGTGGTTTCGATACGTCGCTTAGTGCTTGACTATGCTATGCATGTTTAGCTGCGCAGTG
TCGTGCTGTGCTACATTCAGTCATCCAGCGCTGCACAGTGTTGTGCATGATTGACAAAAATACCCCCT
CGGCCATGCGCACCTTGCCCCGTCCAGCCCACGTCTACTTCCTTCAGATGTTCTGTGTACACCAACCC
CCTGCTCGATCACAGAAATATCACAGCACGAACTGGACTGTTCCTTGCTTTCAGCATGTGCGCGCGCG
CGCCTGTGTGTGCATATGTGTATATGTGTGTCGAAATGTGGCGGCAATATCGCTTTTTCTCTATTCCG
AGGGAACAGCATCTGCCAAGGAGCACCCCTGTGTCTCATTTTCGCAAGTGTCCTTGTAACCCGTCCCA
ACCCAAACCCACTCCGCGTCCCCTCATGGACCCCCCCCCACGCGCCATGGGCCCCTGGCCACATCCTG
ATACAACCAAAGCTTGCCCTCTTGAGGTGCTATATGACCTGCTTTTCACTGACCCCCTACAACCCCAA
AACTCACATCCTACGCGCCCTCCTCTTTTTCCCCCGCGCCCAGGTGCGTGTCCCGGTCCCCTTCCAGC
TACCCATGGCGCGTGACCGTCGCCAACGTTTCCGCGGTCACCATCTCTGGCGGCGCTGGCGAGCGCGT
GTGCCTGAAGATTTCGGTCGACAAC**GCGGCCGC**TGCCACCTGCAACAACGGCCTGGGCGGGTGCTGCT ***Not*I**
CGGATGGACTGGAGAAGGTC**GAGCTC**TTTGCTAGTGAGTACCTCAGCGTTGCGGCGGCAATTTGTGTG ***Sac*I**
AATGTGTGTTTGTGTGTGTGTGGGGGGGGGGAATGGGGGGTGGGGGTGTAATGGCGGCGCGGGAGATT
GGGATCACGGGACTGACACTACCATCAGATGGCTTTGGGGGAGAGGATGGGATTTTCGGGTGAACTGG
AACAAGGATGTGTGTGTGTGTGTATGTGTGTATGTGTGTTGTTCATGCTGCTCTAGCAGCTGCGGAAA
CAGCTCCTGGGTGATGGGTGGCTTCCTAGGCACTACAGCTACGGGCTATGGCGAGCCCGTCATAGCAG
CCTGCTGGGAAGCCGATGTGGTGCGTAACGTTGGTCGCTTAGGAGGGGTCGTGGAGGGGTCGTGGAGG
GGTCGTGGAGGGGTCGTGGAAGTATCGATGTTGCGTTTGCGGTGACCCAGCGTGAAGAATAACAAATG
CCAACAGTGTCGGAGTGTTATCAGGTGCAAAGTCCTCCGCAGTGCTGATGCCTCCTCTTCAGCAGCAG
CCTGCCCTACGCGATTACACGGTGGTTGAAGTGGTTGCTGGTTGGCGCCGCCAGCATTATATTTTAAC
CACGTCAGGGCCTGATGCGCCGTGGGTTTAGGAAGCAGCTTCTCGCCTGAAGGGAGGGGGAGACGGGT
TCTGGGTTGGTTTGGTTCTGGTGCCCGCTAGGGTGAGAATTGCTGAAGGGCTGACGTGTACCAGGTCA
TATGTACCCTAGTGGCGCGAAGTAAACAGACGAGCTAGGGAACATAACAATAACAACAGCAAATACAA
TAAGCTTTAAAACGATCTATTGATATGCGCTGCATGTGCGTGCGTATGTATGTGTGTATACGGCGATG
CGAGTACGGCATCTTTCCATACTCTGGTCTTTAATCTCACCCCTGAAACACACCACCTTCGTTTGCGC
GTGCATCCATGCGTGTGTGGTGTGGGCTGGCGTGGCCTAGTAAGTGGAAATGATTTCAAAGCCAAAAC
AAGCTACCGGGATAGCATCAGTCTCTTCAATCATTCACCCGGTCTCCGCGTGTGGCGCTTGGGGGTAT
GTTCCCACTGCCGCCACTGTCACCTGCTGAGCTGTCGGCATGTCGTAATAACGCCTCTGCACGCCAGC

CCGTGGTATCGTTTTCCG**GGATCC**CGCTGTCGGGTCTAAGAATGGCTTCCGTTGCTGGCGCTGCTCGC	***Bam*HI**
TCGAACGCGAGACCAAGTTAACTTTTACTCCTGGAGCAGAAGGTTTAGTAAAACAAGCTATTGCTGAA	
TATTTAGAA**GAATTC**AAATCAAGTGCTAAAGCTGCTTAATTAGTAATAATTTATATAATTTGGTGTCC	***Eco*RI**
ACAACATACATAGCTGCCGGTTGCAACCACATCTACACCCACATATGTCTGTGCGCGTGCGTGTTGCA	
CCTTGGGCTGCTTATGTGCTACCCTACGAATGTATTAACCTAGAGCCCCATTTACATGTGCTCAACCA	
TGCCTACCAACACAACACCAACACATTTCCCCTTCGCACCCGTTCTCTGTTCGAACCCCACAGACGGC	
AAGTGCAAGGGCTCCATTTTGCCGTTCACTCTGTCCAACACCGCGGAGATTAGGTCGTCATTCTCCTG	
GGACTCCACTCGCCCAGTCCTCAAATTCACCCGCCTGGGCCTCACCTATGCTCAAGGTGTCGCCGGCG	
GCAGCCTCTGCTTCAACATCAAGGGTGCCGGCTGTACCAAGTTTGC**AGATCT**CTGTCCTGGCCGCGGC	***Bgl*II**
TGCACCGTCGCGGTATTCAACAACCCCGACAACACATGTTGCCCGCGGGTCGGCACCATCGCG**GGTAC**	***Kpn*I**
**C**GGCGGAGGCGGTGGCATGAGCAAGGGCGAGGAGCTGTTCACCGGCGTGGTGCCCATCCTGGTGGAGC	gly5
TGGACGGCGACGTGAACGGCCACAAGTTCAGCGTGAGCGGCGAGGGCGAGGGCGACGCCACCTACGGC	
AAGCTGACCCTGAAGCTGATCTGCACCACCGGCAAGCTGCCCGTGCCCTGGCCCACCCTGGTGACCAC	
CCTGGGCTACGGCCTGCAGTGCTTCGCCCGCTACCCCGACCACATGAAGCAGCACGACTTCTTCAAGA	
GCGCCATGCCCGAGGGCTACGTGCAGGAGCGCACCATCTTCTTCAAGGACGACGGTAACTACAAGACC	
CGCGCCGAGGTGAAGTTCGAGGGCGACACCCTGGTGAACCGCATCGAGCTGAAGGGCATCGACTTCAA	*yfp*
GGAGGACGGCAACATCCTGGGCCACAAGCTGGAGTACAACTACAACAGCCACAACGTGTACATCACCG	
CCGACAAGCAGAAGAACGGCATCAAGGCCAACTTCAAGATCCGCCACAACATCGAGGACGGCGGCGTG	
CAGCTGGCCGACCACTACCAGCAGAACACCCCCATCGGCGACGGCCCCGTGCTGCTGCCCGACAACCA	
CTACCTGAGCTACCAGAGCAAGCTGAGCAAGGACCCCAACGAGAAGCGCGACCACATGGTGCTGCTGG	
AGTTCGTGACCGCCGCCGGCATCACCCTGGGCATGGACGAGCTGTACAAG**GGTACC**TAAAGCGTTTCG	***Kpn*I, stop**
CATGACGATGCAAAAAAAAGGGTGGTGCAGCGCAACACCCCCCCGTGGGTGTGTGGGTGTGTGTGCCC	
GCGCAAGAAGATTGTGCGTGAGTGGATGTGCGCGGATGTGGGGGGGAAAACCGCCAGTCCCTTTCTTA	
TATGCTGCTACACATGACTGAGGTTGCTGCCGGGGAGGCGGAGGAGGCTTGTGGGTGTGGCGTCGGCG	
TCGATGCCAAGTCACCGCTGTGCCACTGCTGCGGCGGGCTTGGATAGCAGCAGCAGCGGCGCAGCAGC	
AGCAGCATCCGCAGCATCAGCAGCATAGGGACGCATGCATGCCTTTAGCCGGAATGGGAGGGCTGCGT	
GCTGCTTTTGCAACCGCAGGGGTGAAAGTAGGTAGTCCTGCATAAATGATTAAGCACTGGTCAAATGT	
CGCTGAGATGAACATAACACGATTATATATGATATGATGCGCGACATGATTGTTGGATGCGGCCAGTA	
CTTTTTTCCGCTGCGCAGGTGTCAGTGTTGCACTTGGGTTTGATCCAGTTACGGGCCTCTCGGCGTCT	
ACCAAACGCAAATTGTCGTTAATTGATATTCTCTTGCTTTTTGCGCATCTCGTCAAGTGAGGGCAGCG	
TCCCCCTCCTGTGCTGCTGCACTTGACGTGGCTGTTGGAATGTTCCGTGCATGGCCGCTGGCCCGAAC	
CATTTGTAGTTGAGTGACTACTGCGCTAACATTCAGTTATTTCGTAATAATCACCATCATAATTATCA	
TCATCGCTACCTGCCTATGGCCGGGAAGTTGAGCAGGTTTTAACGTTTTACGTTCAATCCCGATGGCC	
CACGTCACGTGTAGCAGGCCTTTGCGTTCCCGCTCTCTTGTAACTTATTTCTCATA**GATATC**AAATTT	***Eco*RV**
GGCTCATCTTTGCAGTGGAGTATTCCTACAAATAGAGGTGCATCTGCCAGATTTGGCAAATAGAGGTG	
CATCTGCCAGATTTGGCCGGTGGGGGACCTTATTAACCGTCGTTAACCA**GGATCC**	***Bam*HI**

The vector pSSG-YFP carries a 8.8-kb fragment of *V. carteri* genomic DNA containing the *ssg185* gene including its 6 introns, a short linker sequence, which codes for 5 glycines (Gly5) and the coding sequence of *yfp* (mVenus). The chimeric gene is controlled by the original *ssg185* 5' and 3' flanking sequences, including the promotor region. The corresponding gene entry in the current *Volvox carteri* genome version 2.1 (Prochnik et al., 2010) in Phytozome 12.1.6 (Goodstein et al., 2012) is Vocar.0002s0564. The *yfp* (mVenus) gene has been codon-adapted for *C. reinhardtii* (Lauersen et al., 2015) but also works well in *V. carteri* (Tian et al., 2018; von der Heyde and Hallmann, 2020). Artificial *Kpn*I and *Bam*HI sites have been introduced to facilitate cloning. The background colors indicate the following sequence features: gray, *ssg185* promoter region, green, 5' and 3' UTRs of *ssg185*; blue, coding sequences of *ssg185*; turquoise, sequence coding for 5 glycines (Gly5); yellow, coding sequence of *yfp* (mVenus). Start and stop codons are highlighted (violet font). A previous sequence gap, which we closed, is also indicated (blue font). Relevant restriction sites are marked (bold, underlined).

References

Goodstein, D. M., Shu, S., Howson, R., Neupane, R., Hayes, R. D., Fazo, J., Mitros, T., Dirks, W., Hellsten, U., Putnam, N. et al. (2012) Phytozome: a comparative platform for green plant genomics. *Nucleic Acids Res.* **40**, D1178-1186.
Lauersen, K. J., Kruse, O. and Mussgnug, J. H. (2015) Targeted expression of nuclear transgenes in *Chlamydomonas reinhardtii* with a versatile, modular vector toolkit. *Appl. Microbiol. Biotechnol.* **99**, 3491-3503.
Prochnik, S. E., Umen, J., Nedelcu, A. M., Hallmann, A., Miller, S. M., Nishii, I., Ferris, P., Kuo, A., Mitros, T., Fritz-Laylin, L. K. et al. (2010) Genomic analysis of organismal complexity in the multicellular green alga *Volvox carteri*. *Science* **329**, 223-226.
Tian, Y., Gao, S., von der Heyde, E. L., Hallmann, A. and Nagel, G. (2018) Two-component cyclase opsins of green algae are ATP-dependent and light-inhibited guanylyl cyclases. *BMC Biol* **16**, 144.
von der Heyde, B. and Hallmann, A. (2020) Targeted migration of pherophorin-S indicates extensive extracellular matrix dynamics in *Volvox carteri*. *Plant J.* **6**, 2301–2317.
