# Supplementary material for: Cell Type-Specific Pherophorins of Volvox carteri Reveal Interplay of Both Cell Types in ECM Biosynthesis
Source: Cells. 2022 Dec 29;12(1):134. doi: 10.3390/cells12010134 (PMC9818292; doi:10.3390/cells12010134)
Supplement: Supplementary file 1 [file cells-12-00134-s001.zip › Supplemental Figure S3.pdf]

Supplemental Figure S3

HR domains in *V. carteri* and *C. reinhardtii* pherophorins

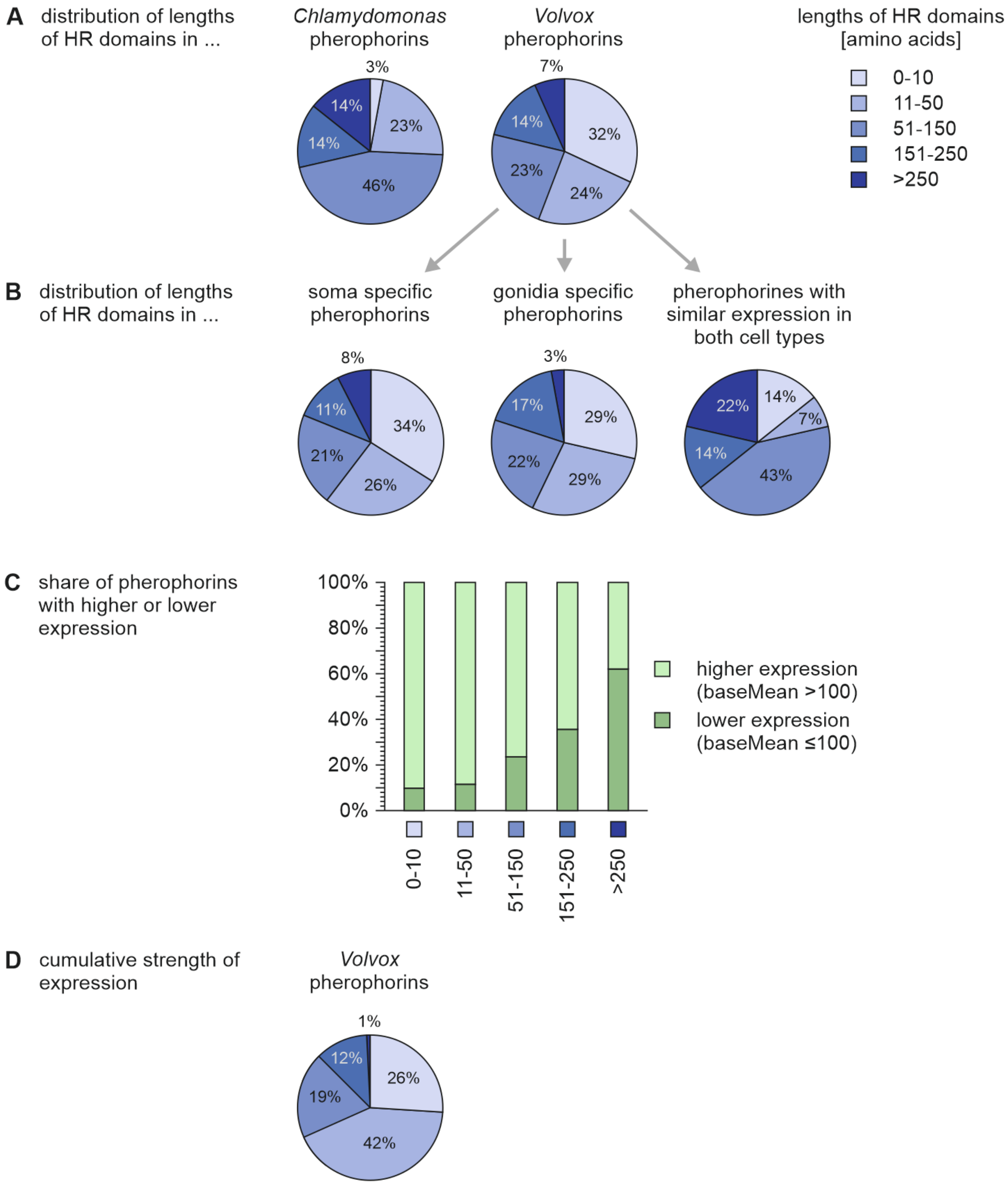

The analysis of proline-rich regions was performed using the sequences of pherophorins as shown in Supplemental Table S1. The most accurate gene model was chosen from the current annotation of the *V. carteri* genome (v2.1) (Prochnik et al., 2010) in Phytozome 12 (Goodstein et al., 2012), from older annotations (v2.0 and v1.0) or from sequences that were deposited in sequence databases in connection with the published characterization of a certain pherophorin (Supplemental Table S1). **A** The sequences of pherophorin-related proteins of *V. carteri* (N=118) and *C. reinhardtii* (N=35) were analyzed for the lengths of their HR-domains, the

corresponding pherophorins were classified accordingly and the share of each class was determined. **B** Among all *V. carteri* pherophorins with reliable expression data (N=103) based on RNA-sequencing data (Klein et al., 2017), we further distinguished between pherophorins that are specifically expressed in somatic cells (N=53) or in gonidia (N=35) or show similar expression in both cell types (N=14). Again, the corresponding pherophorins were classified according to the lengths of their HR-domains and the share of each class was determined. **C** The *V. carteri* pherophorin genes were divided in two groups with higher (baseMean >100) or lower expression (baseMean ≤100) and the corresponding pherophorins were classified according to the lengths of their HR-domains. Within each HR-domain class the share of pherophorin genes with higher or lower expression was determined. **D** Cumulative intensity of expression of all *V. carteri* pherophorin genes, subdivided in groups according to the lengths of the HR domains of the corresponding pherophorins. The cumulative intensity of expression reflects the summed up average baseMean values of the pherophorin genes.

References

Goodstein, D. M., Shu, S., Howson, R., Neupane, R., Hayes, R. D., Fazo, J., Mitros, T., Dirks, W., Hellsten, U., Putnam, N. et al. (2012) Phytozome: a comparative platform for green plant genomics. *Nucleic Acids Res.* **40**, D1178-1186.

Klein, B., Wibberg, D. and Hallmann, A. (2017) Whole transcriptome RNA-Seq analysis reveals extensive cell type-specific compartmentalization in *Volvox carteri*. *BMC Biol* **15**, 111.

Prochnik, S. E., Umen, J., Nedelcu, A. M., Hallmann, A., Miller, S. M., Nishii, I., Ferris, P., Kuo, A., Mitros, T., Fritz-Laylin, L. K. et al. (2010) Genomic analysis of organismal complexity in the multicellular green alga *Volvox carteri*. *Science* **329**, 223-226.
