# Supplementary material for: Cell Type-Specific Pherophorins of Volvox carteri Reveal Interplay of Both Cell Types in ECM Biosynthesis
Source: Cells. 2022 Dec 29;12(1):134. doi: 10.3390/cells12010134 (PMC9818292; doi:10.3390/cells12010134)
Supplement: Supplementary file 1 [file cells-12-00134-s001.zip › Supplemental Figure S4.pdf]

Supplemental Figure S4

Schematic structure of the *phG* gene, *phG* mRNA and pherophorin-G protein

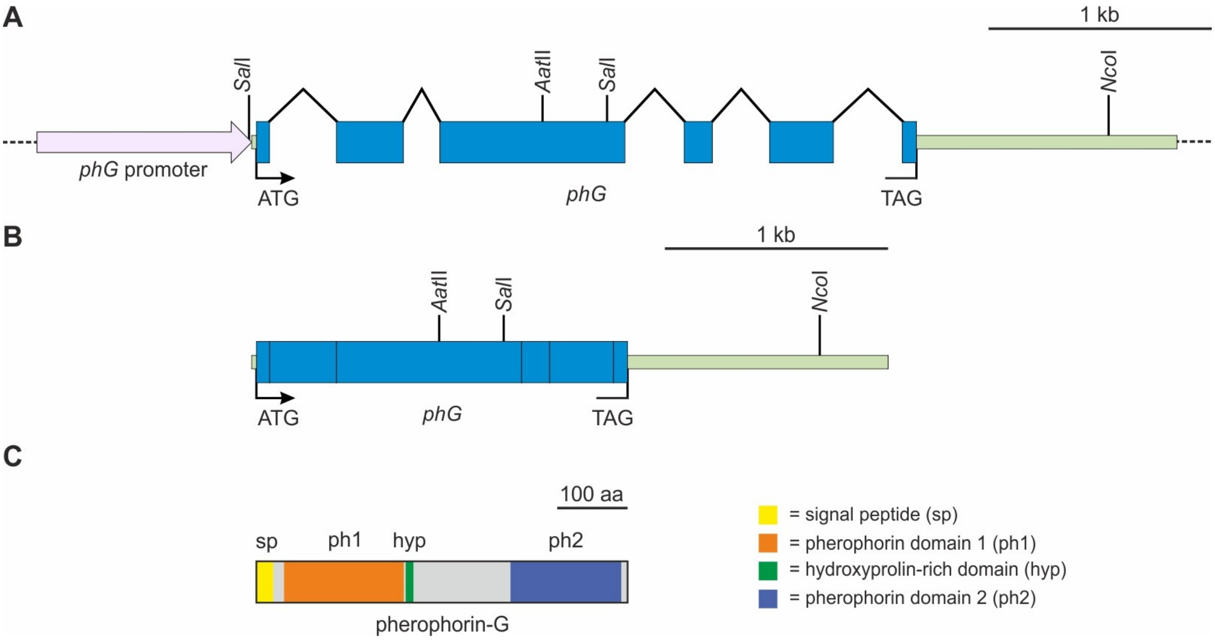

A The sequence schematized here corresponds to the 5244-bp genomic fragment utilized in plasmid pPhG-YFP. The *phG* gene (former common name *phV47*) (von der Heyde and Hallmann, 2020), is located on scaffold 1 (nucleotides 2224937 to 2230180) of the *V. carteri* genome version 2.1 (Prochnik et al., 2010) in Phytozome 12.1.6 (Goodstein et al., 2012) and has the gene ID Vocar.0001s0298. The start codon is at nucleotide position 2225951-2225953 on the forward strand. The gene structure is indicated as follows: Coding sequences are represented by blue squares, intron sequences by carats, UTRs by green bars and the promoter region by a pink arrow. Start (ATG) and stop (TAG) codons are highlighted. **B** Structure of the *V. carteri phG* mRNA. Sequence features are as indicated in A. The coding sequence (blue squares) totals 1,581 nucleotides. The 5' UTR is 14 bp in length, while there is a quite long 3' UTR of 1168 bp. The complete mRNA is 2,763 nucleotides in length. **C** Structure of the *V. carteri* pherophorin-G protein. The polypeptide comprises 526 amino acids and the calculated molecular weight amounts to 57.3 kDa. As pherophorin-G is an extracellular protein, it possesses a cleaved N-terminal signal peptide (sp) of 24 amino acids. In the mature protein three domains can be identified: an N-terminal pherophorin domain (ph1) with an E-value of 1.9e-15, a hydroxyproline-rich domain (hyp) in the middle (Hallmann, 2003) and a C-terminal pherophorin domain (ph2) with an E-value of 1.8e-28. The pherophorin domains where identified by blasting the Pfam database (El-Gebali et al., 2019) using the hmmscan function (Potter et al., 2018). The Hydroxyproline-rich domain consists of 82% (hydroxy) prolines.

References

El-Gebali, S., Mistry, J., Bateman, A., Eddy, S. R., Luciani, A., Potter, S. C., Qureshi, M., Richardson, L. J., Salazar, G. A., Smart, A. et al. (2019) The Pfam protein families database in 2019. *Nucleic Acids Res.* **47**, D427-D432.
Goodstein, D. M., Shu, S., Howson, R., Neupane, R., Hayes, R. D., Fazo, J., Mitros, T., Dirks, W., Hellsten, U., Putnam, N. et al. (2012) Phytozome: a comparative platform for green plant genomics. *Nucleic Acids Res.* **40**, D1178-1186.
Hallmann, A. (2003) Extracellular matrix and sex-inducing pheromone in *Volvox*. *Int. Rev. Cytol.* **227**, 131-182.
Potter, S. C., Luciani, A., Eddy, S. R., Park, Y., Lopez, R. and Finn, R. D. (2018) HMMER web server: 2018 update. *Nucleic Acids Res.* **46**, W200-W204.
Prochnik, S. E., Umen, J., Nedelcu, A. M., Hallmann, A., Miller, S. M., Nishii, I., Ferris, P., Kuo, A., Mitros, T., Fritz-Laylin, L. K. et al. (2010) Genomic analysis of organismal complexity in the multicellular green alga *Volvox carteri*. *Science* **329**, 223-226.
von der Heyde, B. and Hallmann, A. (2020) Targeted migration of pherophorin-S indicates extensive extracellular matrix dynamics in *Volvox carteri*. *Plant J.* **6**, 2301–2317.
